# Spatial–seasonal characteristics and critical impact factors of PM_2.5_ concentration in the Beijing–Tianjin–Hebei urban agglomeration

**DOI:** 10.1371/journal.pone.0201364

**Published:** 2018-09-20

**Authors:** Tianhang Huang, Yunjiang Yu, Yigang Wei, Huiwen Wang, Wenyang Huang, Xuchang Chen

**Affiliations:** 1 School of Public Policy and Management, University of Chinese Academy of Sciences, Beijing, China; 2 International Business School, Shanghai Lixin University of Accounting and Finance, Shanghai, China; 3 School of Economics and Management, Beihang University, Beijing, China; 4 Beijing Key Laboratory of Emergency Support Simulation Technologies for City Operation, Beijing, China; 5 School of Economics and Management, University of Chinese Academy of Sciences, Beijing, China; University of Maryland at College Park, UNITED STATES

## Abstract

As China’s political and economic centre, the Beijing–Tianjin–Hebei (BTH) urban agglomeration experiences serious environmental challenges on particulate matter (PM) concentration, which results in fundamental or irreparable damages in various socioeconomic aspects. This study investigates the seasonal and spatial distribution characteristics of PM_2.5_ concentration in the BTH urban agglomeration and their critical impact factors. Spatial interpolation are used to analyse the real-time monitoring of PM_2.5_ data in BTH from December 2013 to May 2017, and partial least squares regression is applied to investigate the latest data of potential polluting variables in 2015. Several important findings are obtained: (1) Notable differences exist amongst PM_2.5_ concentrations in different seasons; January (133.10 mg/m^3^) and December (120.19 mg/m^3^) are the most polluted months, whereas July (38.76 mg/m^3^) and August (41.31 mg/m^3^) are the least polluted months. PM_2.5_ concentration shows a periodic U-shaped variation pattern with high pollution levels in autumn and winter and low levels in spring and summer. (2) In terms of spatial distribution characteristics, the most highly polluted areas are located south and east of the BTH urban agglomeration, and PM_2.5_ concentration is significantly low in the north. (3) Empirical results demonstrate that the deterioration of PM_2.5_ concentration in 2015 is closely related to a set of critical impact factors, including population density, urbanisation rate, road freight volume, secondary industry gross domestic product, overall energy consumption and industrial pollutants, such as steel production and volume of sulphur dioxide emission, which are ranked in terms of their contributing powers. The findings provide a basis for the causes and conditions of PM_2.5_ pollution in the BTH regions. Viable policy recommendations are provided for effective air pollution treatment.

## Introduction

The rapid urbanisation, industrialisation and modernisation that China has undergone have improved household income and living standards but have also led to extensive and severe issues of air pollution. In particular, air pollution has become a major environmental challenge for most Chinese cities [1–5]. Various particulate matters (PMs) are recognised as key air pollutants that have serious adverse impacts on human health. Scientific reports indicate that excessive exposure to high PM concentrations reduces the expected human lifespan by 1–5.5 years [6]. In 2013, the WHO identified PMs as the leading cause of human cancer.

Atmospheric PM can be divided into several categories on the basis of their aerodynamic diameters, namely, total suspended PM, PM with a particle size below 10 μm (PM_10_) and fine PM (PM_2.5_) [7–8]. Fine PM_2.5_ with a diameter less than 2.5 mm is widely recognised as one of the most detrimental airborne PMs because small particle pollutants can enter the lungs and alveolar macrophage through breathing [9]. Long-term exposure to PM_2.5_ leads to high incidences of cardiovascular and respiratory diseases and chronic bronchitis cases. According to the Global Burden of Disease 2010 Comparative Risk Assessment, high PM_2.5_ concentrations have caused 3.1 million deaths worldwide [10]. Despite the apparent reductions on ‘traditional pollutants’ (e.g. NO_2_ and SO_2_) in recent years, PM_2.5_ has become a major air pollutant that threatens human morbidity and mortality in developing countries [11–15]. Severe and frequent smog and haze weather (high PM_2.5_ concentration) have also become major barriers for China in attracting foreign investments and talents [16]. Although PM_2.5_ has been incorporated in the official monitoring and appraisal system in Europe and North America for more than 30 years [17–19], China only included PM_2.5_ as a routine monitoring indicator in 2013. Numerous studies have focused on PM_2.5_ pollution in developed and emerging economies [20–26]. Some studies showed that 20%–30% of PM_2.5_ in Chinese cities originated from coal combustion [20, 22, 27, 28]. For example, Aldabe et al. (2011) [29] investigated the chemical compositions and source apportionments of PM_2.5_ in North Spain, Cesari et al. (2018) [26] studied seasonal variability in Southern Italy and Lonati et al. (2005) [30] examined PM_2.5_ composition in Milan, Italy. Ram et al. (2008) [31] confirmed that the contribution of secondary organic carbon to PM_2.5_ is relatively high in developing countries, such as India. Donkelaar et al. (2010) [32] developed the first global PM_2.5_ distribution map based on satellite data. The data indicated that most polluted regions are mainly located in North Africa and East Asia. In conclusion, relevant studies show that chemical compositions and source apportionments of PM_2.5_ significantly vary in different regions and countries.

At the end of the last century, China officially incorporated PM_10_ and PM_2.5_ in its official environmental monitoring and appraisal system [33]. Several studies showed that Beijing’s annual average PM_10_ mass concentration maintained a consecutive upward trend, increasing from 162 μg/m^3^ to 166 μg/m^3^ from 2000 to 2002 (Beijing Environmental Protection, 2004, 2005) [34, 35]. Beijing’s annual average PM_2.5_ concentration, which was measured at five sites, was relatively high in 2000, varying from 87.6 μg/m^3^ to 111.9 μg/m^3^ [36]. From January 2004 to December 2012, high levels of PM_10_ and PM_2.5_ were obtained in Beijing, with annual mean values of 138.5 ± 92.9 μg/m^3^ and 72.3 ± 54.4 μg/m^3^, respectively [37]. The Beijing–Tianjin–Hebei (BTH) region is the largest urban agglomeration in North China and is the core economic zone in China. According to China Environmental Status Bulletin 2015 [38], 70 cities at the prefecture level and above in BTH and surrounding regions experienced 1710 episodes of severe pollution in 2014, which accounted for 41% of the national total [39].

Mainstream literature on China’s air quality has focused on the chemical compositions of air pollutants, namely, elemental constituents, organic compounds and major inorganic ions [27, 28]. Several studies based on remote sensing and modelling techniques revealed that PM_2.5_ concentrations in China’s urban areas are significantly higher than in rural areas [40, 41]. Zhang et al. (2013) [22] indicated that motor vehicle ownership is the key contributing factor to Beijing’s air pollution, accounting for 63% of the carbonaceous components on PM_2.5_, whereas coal combustion is the main air pollutant source in Tangshan, accounting for 30% of PM_2.5_ compositions. Ma and Zhang (2014) [16] investigated the PM_2.5_ distribution characteristics in China from 2001 and 2010 based on satellite data developed by Battelle Memorial Institute. Their study confirmed that the spatial aggregation effects of PM_2.5_ are apparent in China. Specifically, highly polluted areas are mainly concentrated in the BTJ region, Yangtze River Delta (YRD) and the linking zones.

Several studies have focused on the PM_2.5_ characteristics in the BTH region. Using data from 1497 station-based monitoring sites, Shen and Yao (2017) [42] investigated the effects of demographic and economic factors on PM_2.5_ concentration in four urban agglomerations, namely, BTH, YRD, Pearl River Delta (PRD) and Chengdu–Chongqing (CC). The estimated results indicated that a high correlation exists amongst population density, economic affluence and PM_2.5_ concentration. Geography is another important determining factor for PM_2.5_ concentration because high altitudes are usually associated with high PM2.5 concentrations. Zhou et al. (2017) [43] investigated the impact of economic and ecological factors on PM_2.5_ concentrations by using a two-stage distribution lag model. Their estimated results indicated that the emission of atmospheric pollutants causes hysteresis effects on PM_2.5_ concentrations. Specifically, coal consumption, industrial exhaust, value-added from heavy pollution industry and the ownership of ‘yellow label car’, which are heavy-polluting vehicles, are the key sources of PM_2.5_ pollutant emissions. On the basis of panel data in the last 10 years, Li and Yin (2017) [44] utilised a panel threshold model to investigate the nonlinear changing patterns between socioeconomic development and PM_2.5_ concentration. The study confirmed that the development of the manufacturing and construction sectors and the growth of automobile volumes aggravate PM_2.5_ pollution when the value-added of the tertiary industry is below the threshold value of 6,080 billion. Moreover, the development of the second and third industries was noted to be an effective roadmap to alleviate PM_2.5_ pollution when the value-added of the tertiary industry exceeds 6,080 billion. Su and Zhong (2015) [45] analysed the natural and man-made contributing factors of PM_2.5_ in nine key economic circles in China, such as BTH, YRD, PRD and CC, by using a factor analysis method. They concluded that the effects of human activities are more significant than those of natural factors, and industrial activities are the important contributing factors of PM_2.5_.

These findings also stimulated another key question regarding the seasonal and spatial characteristics and critical impact factor of PM_2.5_ concentrations. However, considering the insufficient long-term and large-scale PM_2.5_ concentration data [46], especially the lack of real-time monitoring data [47], few studies have quantitatively estimated these factors in BTH regions [5, 48]. Although the BTH urban agglomeration is recognised as one of the most severely polluted regions in China [24, 49], analyses on the seasonal and spatial characteristics of PM2.5 spanning a long period are limited [50–52].

The present study aims to estimate the PM_2.5_ concentration characteristics in the BTH urban agglomeration. Specifically, the key objectives of this study are as follows: 1) to investigate the relationships between seasons and PM_2.5_ concentrations (i.e. seasonal variation characteristics), 2) to evaluate the spatial distribution of PM_2.5_ concentrations in different cities and 3) to investigate the critical impact factors of PM_2.5_ concentrations. Real-time monitoring data spanning December 2013 to May 2017 are collected from 80 atmospheric physics observation points by the China Meteorological Administration. Several studies have investigated Beijing’s PM_2.5_ concentration [8, 27, 53]. However, the data sources of these studies were mainly based on satellite images [47], and few studies collected PM_2.5_ real-time monitoring data to significantly improve the estimation accuracy. The current study derives a reliable and accurate estimation on the spatial and seasonal characteristics of PM_2.5_ concentration.

This study provides novel contributions to existing literature as follows: 1) A unique PM_2.5_ data source with a long-time span from December 2013 to April 2017 is used. Real-time monitoring data are consistent with the reliable estimation of satellite data. 2) An array of novel and promising techniques, namely, remote sensing techniques, spatial interpolation and partial least squares (PLS) regression are employed, all of which provide an insightful analysis on PM_2.5_ characteristics in the BTH region. Specifically, spatial interpolation is a powerful approach to replace the traditional inversion method in reflecting PM_2.5_ concentrations at near-ground level [54]. PLS regression is an effective approach to cope with the multicollinearity issue when a large number of independent variables are included in the estimation. 3) Various variables are considered in identifying the critical impact factors of PM_2.5_. Considering that peripheral or distant sources commonly affect the air pollution of a city, this study provides a complete analysis on PM_2.5_ characteristics of a city and a region.

## Data and research methods

### Study area

**Fig 1:**
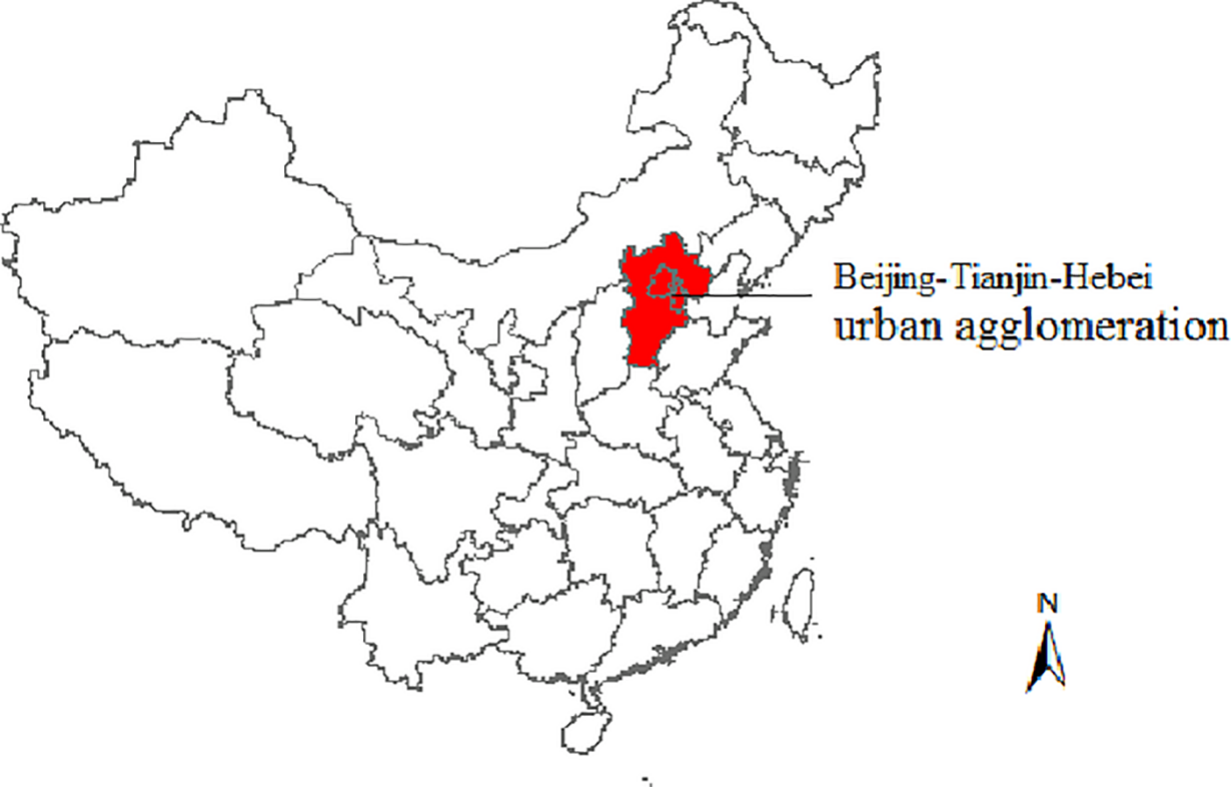
Geographic information of the BTH urban agglomeration.

The study area used is the BTH urban agglomeration, which is also called the Greater Beijing region (Fig 1). This area is located in Northeast China, at longitude 113°04' to 119°53' east and 36°01' to 42°37 ' north. It measures 218,000 km^2^ and had more than 100 million residents as of 2016 (National Bureau of Statistics of China, 2016). The BTH urban agglomeration is the largest urban agglomeration and the most developed economic centre in northern China. Beijing is the political capital, cultural and information centre of China and is one of the largest megacities worldwide, with more than 21 million people and 5.7 million vehicles in 2016 [55]. Given the importance of the Greater Beijing region, severe air pollution has been the leading environmental challenge, with frequent occurrences of fog and haze. Related statistical consensus indicate that the total annual mean values of Beijing’s PM_10_ and PM_2.5_ concentrations from 2012 to 2015were 138.5 ± 92.9 μg/m^3^ and 2.3 ± 54.4 μg/m^3^, respectively [37].

### Data sources

In terms of urban distribution and prefectural boundary, prefectural boundary layers at a scale of 1:4,000,000 are obtained from the National Geomatics Centre of China.

**Fig 2:**
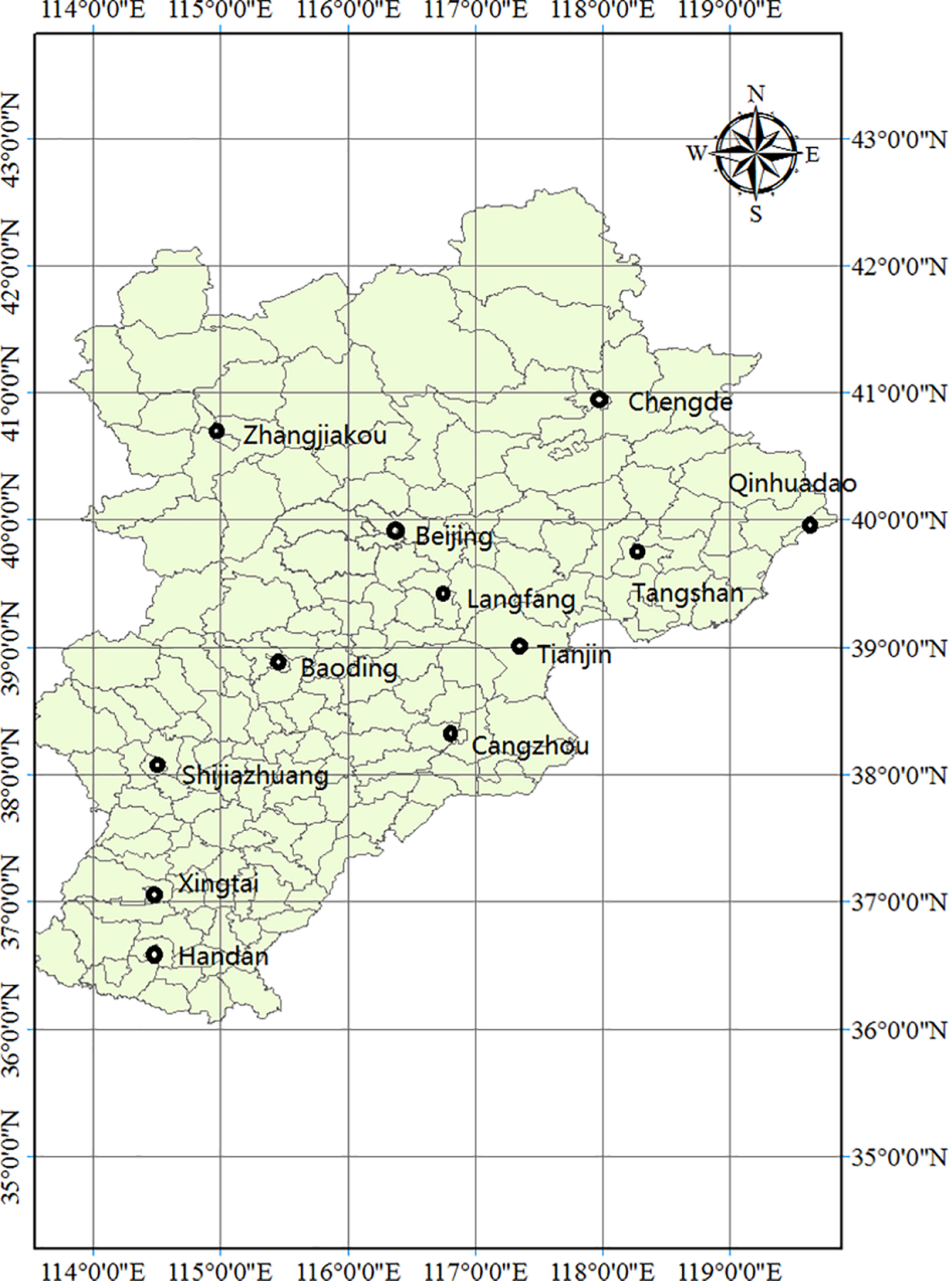
Geographic allocation of the 12 cities in the BTH urban agglomeration.

PM_2.5_ concentration data in the BTH urban agglomeration from December 2013 to May 2017 cover 12 cities, namely, Beijing, Tianjin, Tangshan, Zhangjiakou, Baoding, Handan, Chengde, Qinhuangdao, Xingtai, Cangzhou, Langfang and Shijiazhuang (For details, see Fig 2 and S2 Table).

This monitoring dataset is obtained from the atmospheric physics sites of the 12 cities by the Ministry of Environmental Protection of China. The remote sensing data from the Atmospheric Composition Analysis Group (2016) [56] are initially used in BTH region. Compared with the remote sensing data on PM_2.5_ (Atmospheric Composition Analysis Group, 2016) [56], spatial interpolation has higher accuracy than remote sensing data in reflecting PM_2.5_ concentrations at near-ground level in this study.

**Fig 3:**
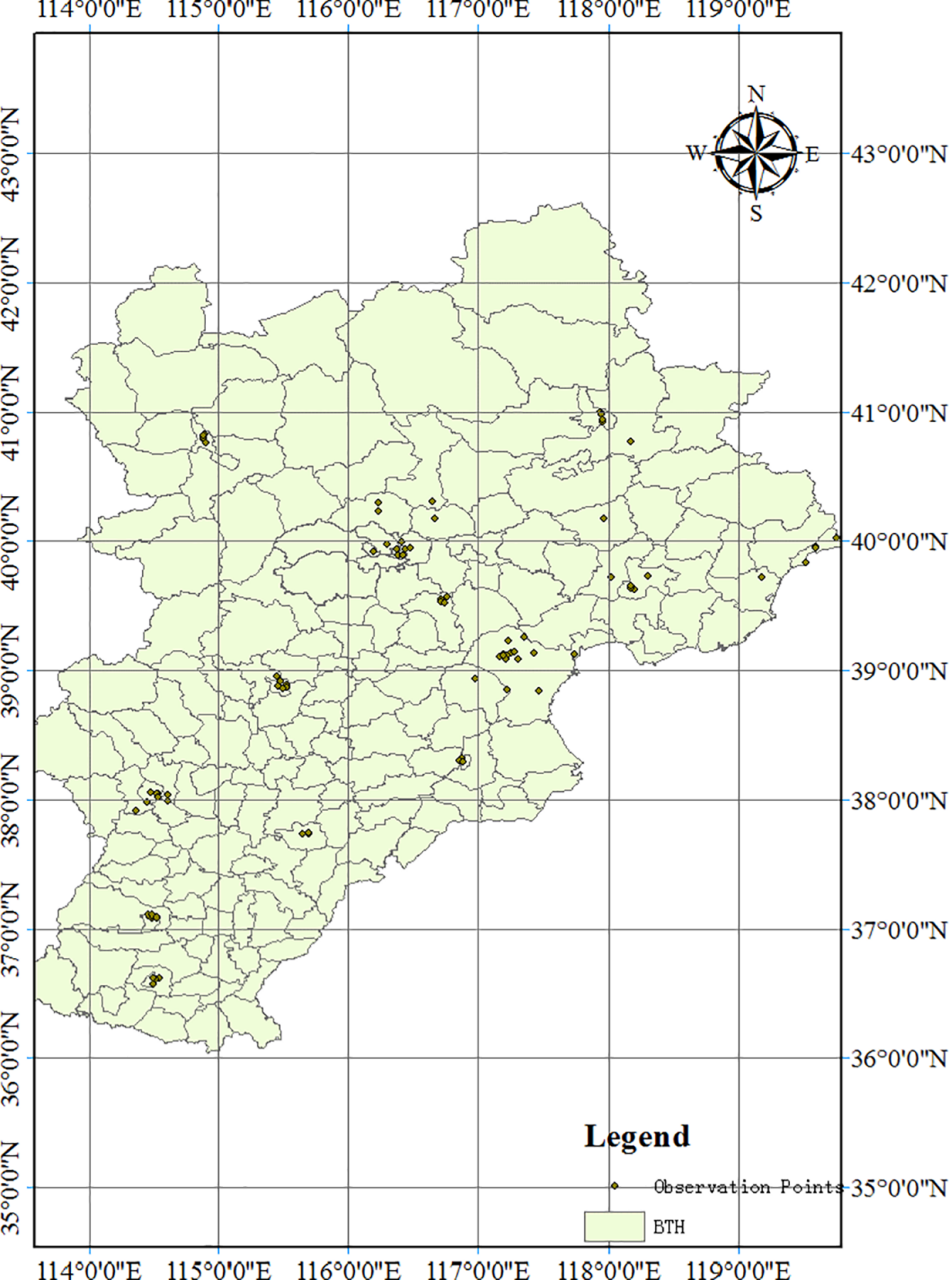
Geographic allocation of 80 atmospheric physics observation points.

These data are measured by 80 monitoring stations distributed throughout the BTH region (Fig 3). Each monitoring station automatically measures monthly PM_2.5_ concentrations. Cangzhou, which is a small city, has three air quality monitoring stations. Other cities have more than five air quality monitoring stations that are distributed from the suburbs to downtown. The annual average of PM_2.5_ concentration for each monitoring station is calculated based on the hourly real-time data. Beijing, Tianjin and Hebei have 12, 15 and 53 atmospheric physics observation points, respectively. Table 1 describes the geographic information on several atmospheric physics observation points. The geographic information of all observation points is shown in S1 Table. In terms of data standardisation, the collected PM_2.5_ values at 2:00, 8:00, 14:00 and 20:00 from different observation points are averaged to derive the daily and monthly PM_2.5_ concentrations of a city. The PM_2.5_ concentration of 12 sites is the average of 80 sites.

**Table 1:** Geographic information on some atmospheric physics observation points.

City	Observation Point	Longitude	Latitude
Beijing	Wanshougong West	116.3747278	39.88565298
Tianjin	Municipal inspection centre	117.1655924	39.10485468
Baoding	Huadian	115.520781	38.8918471

Source: China Meteorological Administration

Table 2 describes an array of PM_2.5_-related variables summarised from an extensive literature review. These variables are tested to identify the critical impact factors for PM_2.5_ concentration. Existing studies on identifying the key contributing factors of PM_2.5_ concentration in China have mainly focused on demographic and economic aspects and other chemical air pollutants. Yang and Chen (2017) [57] used independent variables, namely, coal consumption, cement production, automobile volume, population and gross domestic product (GDP). Lu et al. (2017) [58] incorporated the following variables in their estimation, namely, population density, annual volume of bus passengers, road freight, proportion of secondary industry to overall GDP, volume of SO_2_ emissions and volume of industrial soot emission. Ma and Xiao (2017) [59] considered urbanisation, energy consumption structure[60] and construction areas in their investigation. On the basis of an extensive literature review, 12 potential contributing factors for PM_2.5_ concentrations are identified (Table 2).

**Table 2:** Potential critical impact factors for PM_2.5_ concentration.

Abbreviation	Variable	Unit	Reference
PD	Population density	Persons/KM^2^	[1, 2, 4, 5]
UR	Urbanisation rate	%	[1, 2, 4, 5]
RFV	Road freight volume	10000 tons	[26, 29, 61–63]
SIGDP	Secondary industry GDP	100 millions	[30]
OEC	Overall energy consumption	10000 tons of standard coal	[20, 22, 27, 28, 31, 41]
SP	Steel production	10000 tons	[20, 22, 27, 28, 30, 31, 33]
VOSDE	Volume of sulphur dioxide emission	Ton	[20, 22, 27, 28, 30, 31, 33]
VOISE	Volume of industrial soot (dust) emission	Ton	[20, 22, 27, 28, 30, 31, 33]
CP	Cement production	10000 tons	[20, 22, 27, 28, 30, 31, 33]
MVO	Motor vehicle ownership	10000 units	[29, 33, 61]
RPTV	Road passenger traffic volume	10000 persons	[29, 61]
RNGC	Residential natural gas consumption	10000 cubic metres	[60]

The possible critical impact factors of PM_2.5_ concentration are selected (Tables 2 and 3), discussed and included in the estimation model. Since independent variable data in 2016 and 2017 has yet been published by the statistic consensus, this study used the latest data of 2015 in the PLS model to analyse the critical factors for PM2.5 concentration (For details, see S3 Table).

**Table 3:** Statistic description (2015).

Variable	Std. Deviation	Mean	Minimum	Maximum
PD	264.25	578.89	96.73	870.29
UR	25.77	44.23	7.10	86.51
RFV	12913.84	20361.00	4152.00	38704.00
SIGDP	2134.57	2182.93	445.09	7723.60
OEC	123147.06	39054.27	472.52	430000.00
SP	3587.67	2800.52	31.85	11179.00
VOSDE	57232.31	81347.50	22070.00	214723.00
VOISE	47176.04	63778.92	12987.00	191713.00
CP	784.35	889.35	141.06	2781.00
MVO	165.84	209.15	60.56	561.90
RPTV	13441.63	9055.83	1163.00	49931.00
RNGC	53139.42	22124.42	666.00	189188.00

Data Resource: National Bureau of Statistics of the People’s Republic of China (2016 a, b)

#### Population density

**Fig 4:**
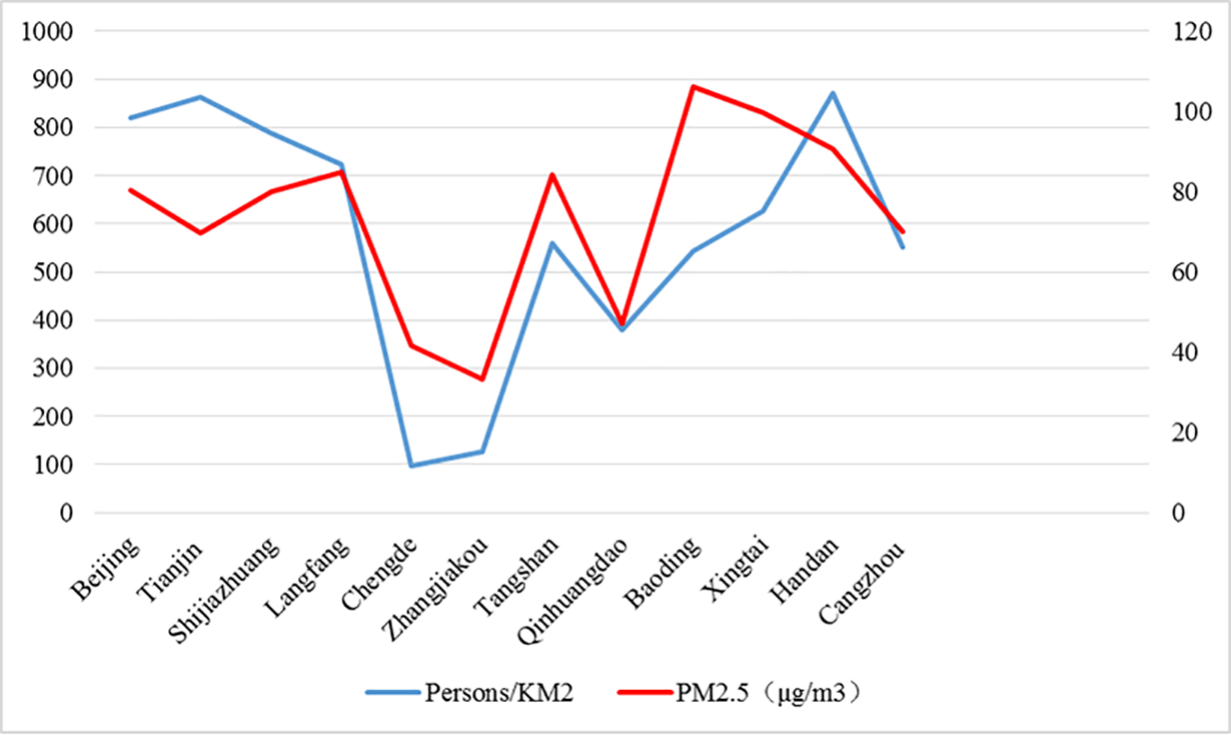
PM_2.5_ concentration and population density per km^2^ in 2015. (Unit: μg/m^3^ refers to the left axis, and Persons/km^2^ refers to the right axis).

A comparison of PM_2.5_ concentrations with population reveals interesting findings that should be considered. Shijiazhuang, which is the most polluted city, features a relatively high population density of 788.14 persons/km^2^. Handan, the second most polluted city, has the highest population density of 870.29 persons/km^2^. Zhangjiakou, which has the best air quality, presents a low population density of 127.46 persons/km^2^. Chengde, with a similar pollution level as Shijiazhuang, also manifests a low population density of 96.73 persons/km^2^ (Fig 4). A common pattern exists in which population density forms a certain positive relationship with PM_2.5_ concentration. However, this pattern is affected by various critical impact factors that lead to certain variations. Therefore, a detailed investigation on critical PM_2.5_ factors should be conducted for a thorough analysis of such particles.

#### Industrial and energy aspects

**Fig 5:**
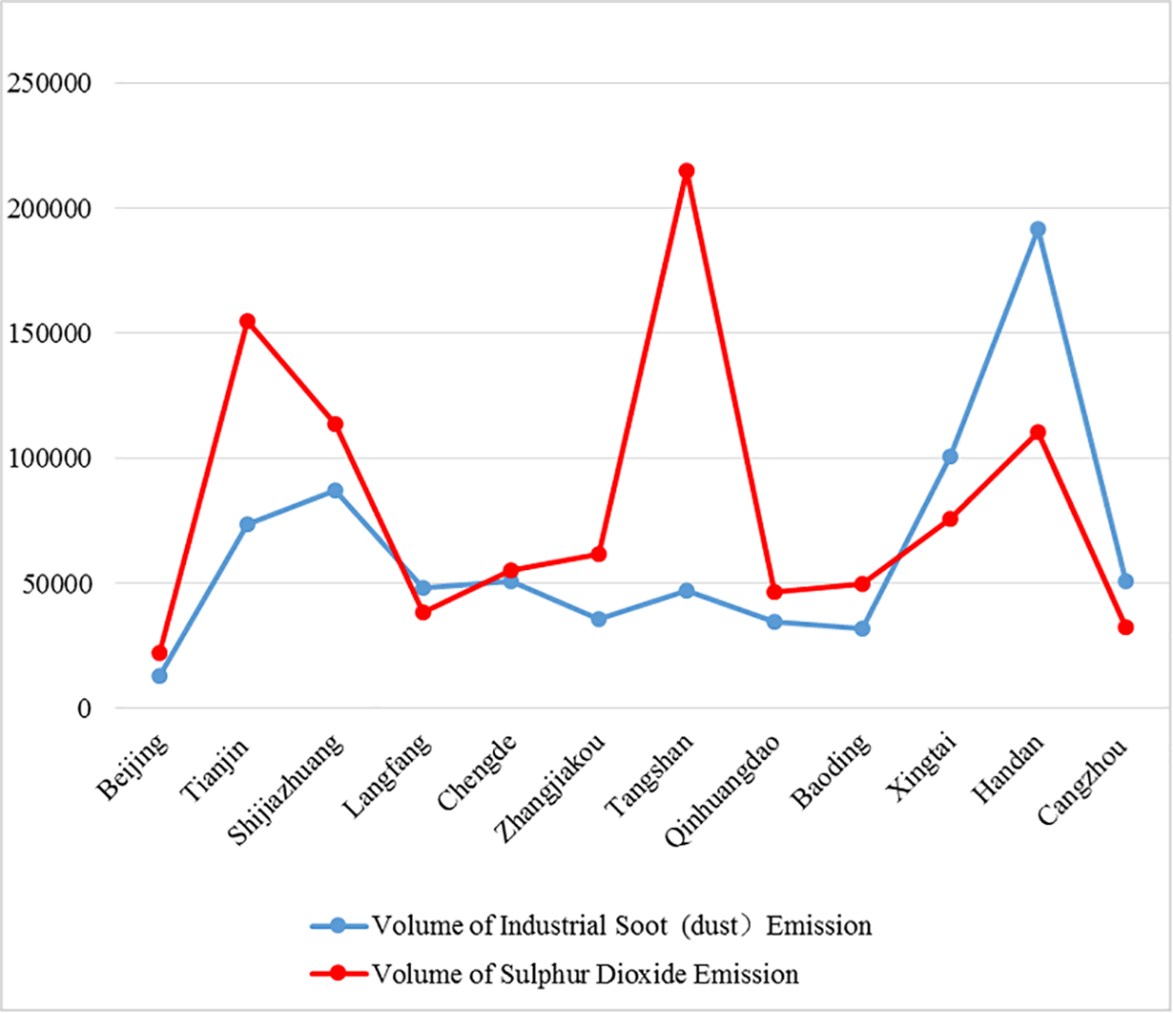
Volume of industrial soot (dust) and sulphur dioxide emissions in 2015 (Unit: Ton).

The estimation results reveal that PM_2.5_ concentration in the BTH urban agglomeration exhibits a distinctive spatial distribution characteristic. Related literature shows that coal combustion accounts for 20%–30% of PM_2.5_ pollution in Chinese cities [20, 22, 27, 28]. In winter, PM_2.5_ concentration is usually high because coal is used as the main energy for winter heating. In summer, the situation significantly differs in the BTH urban agglomeration. For example, motor vehicles account for 63% of the carbonaceous components of PM_2.5_ in Beijing, while coal combustion accounts for 30.3% of PM_2.5_ compositions because it is used as the major energy source for industrial production in the city [22]. Therefore, the present study uses industrial dust and industrial SO_2_ emissions as parameters to investigate the air pollution contributions of heating and industrial development. Tangshan shows the highest volumes of industrial SO_2_ emission, which amounted to 214,723 t in 2016, followed by Shijiazhuang and Handan with 113,652 and 110,193 t, respectively. Xingtai and Handan represent the top two contributors of industrial dust emissions, accounting for 191,713 and 100,738 t, respectively. Fig 5 shows the volume of industrial soot (dust) and sulphur dioxide emissions of 12 cities in BTH.

#### Transportation

Several studies argue that transportation exerts a significant adverse influence on air pollution [29]. On the basis of available data from statistical consensus, ‘passenger and freight volume of highway traffic’ are used as a parameter for measuring PM_2.5_ pollution from transportation. Data suggest that polluted cities are generally associated with high road freight volume. For example, Shijiazhuang, Tangshan and Handan, which are the most polluted cities, are associated with relatively high freight volumes with 3,695,410,000, 363,580,000 and 387,040,000 t, respectively.

### Research methods

**Fig 6:**
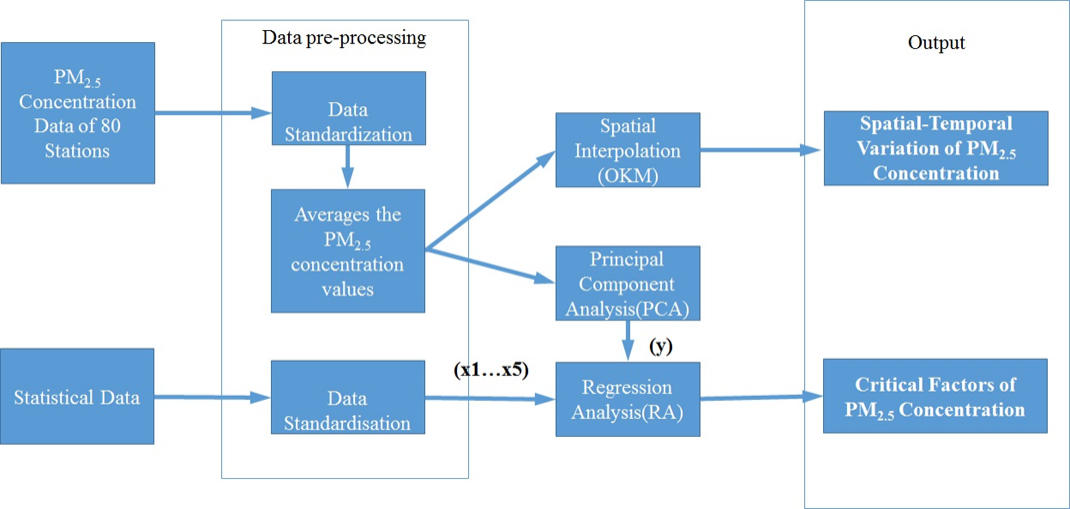
Research framework.

The research framework and main research steps are illustrated in Fig 6.

#### Spatial interpolation

PM_2.5_ concentration is a scalar description of atmospheric state significantly affected by local human activities. Although remote sensing has been improved by techniques such as regional correlations in recent years, several studies indicate that spatial interpolation is a powerful approach to replace the inversion method, leading to higher accuracy than remote sensing data in reflecting PM_2.5_ concentrations at near-ground level [40, 54, 64–67]. To address this limitation, spatial interpolation is employed and the results of the inversion method are considered as references. Interpolation methods used in regional-scale factors include inverse distance interpolation (IDW) and Kriging interpolation method (OKM). OKM is a more widely recognised method for dealing with interpolation points than IDW [22].

This study uses OKM to simulate seasonal variations of PM_2.5_ in the 12 cities of the BTH urban agglomeration. The supporting concept of OKM is that the interpolation results at the target point are the weighted sum of known attribute values of the samples [68]. In the study area, x represents the spatial location of point x. *z(x*_*i*_*)* (i = 1, 2, ⋯, n) represents the property value of sampling point *x*_*i*_ (i = 1, 2, ⋯, n), and annual mean PM_2.5_ concentration is the property value of point x_i_. Then, the interpolation result at target point *x*_*0*_ is *z(x*_*0*_*)*:

$$z(x_{0})=\sum_{i=1}^{n}\lambda_{i}z(x_{i}).$$

Where *λ*_*i*_ (i = 1, 2, ⋯, n) depends on undetermined coefficients. Assuming that the entire study area satisfies the second-order stationary assumption, that is, ‘the mathematical expectation of z(x) exists and is equal to the constant, that is, E[z(x)] = m’, the covariance function of variables z(x) exists and only depends on lag value (h), that is, Cov[*z*(*x*),*z*(*x* +*h*)] = *E*[*z*(*x*)*z*(*x* + *h*)] − *m*^2^ = *C*(*h*).

On the basis of unbiased expectation E[z*(x_0_)] = E[z(x_0_)], E[z*(x_i_)] refers to the spatial variation of PM_2.5_ concentration in BTH by OKM in point x_i_, E[z*(x_0_)] denotes the spatial variation of PM_2.5_ concentration in BTH by OKM in point x_0_, and *z(x*_*0*_*)* is the PM_2.5_ concentration in point *x*_*0*_. We can conclude that $\sum_{i=1}^{n}\lambda_{i}=1$. For regionalised variables that satisfy the second-order stationary conditions, the estimated variance can be calculated using the following formula:

$$\sigma_{E}^{2}=E{\left[z^{*}(x_{0})-z(x_{0})\right]}^{2}-{\left\{E[z^{*}(x_{0})-z(x_{0})]\right\}}^{2}=\sum_{i=1}^{n}\sum_{j=1}^{n}\lambda_{i}\lambda_{j}C_{i,j}-2\sum_{i=1}^{n}\lambda_{i}C_{i,0}+C_{0,0}.$$

To obtain the minimum variance estimation under unbiased conditions, that is, $Min\{Var[z^{*}(x_{0})-z(x_{0})]-2\mu \sum_{i=1}^{n}\left(\lambda_{i}-1\right)\}$.

The weight coefficients should satisfy the following equations:

$$\left\{ \begin{array}{l} \sum_{i=1}^{n}\lambda_{i}Cov(x_{i},x_{j})+\mu =Cov(x_{0},x_{i})\\ \;\sum_{i=1}^{n}\lambda_{i}=1 \end{array}.\right.$$

Then, we can calculate the value of *λ*_*i*_ (i = 1, 2, ⋯, n) and obtain the attribute value z*(x_0_) at sample point x_0_.

1. The degree of correlation between t_1_ and u_1_ should be the maximum.

The two conditions can be summarised as follows:

$$Var(t_{1})\to max$$

$$Var(u_{1})\to max$$

$$Var(t_{1},u_{1})\to max$$

After the first principal components *t*_1_ and *u*_1_ are extracted from X and Y, PLS performs linear regressions of X and Y on *t*_1_. In the PLS estimation, components *t*_1_ and *u*_1_ have typical component characteristics. A significant linear relationship between *t*_1_ and *u*_1_ indicates that X has a notable correlation with Y, and PLS is appropriate for estimating the contribution of X to Y. The algorithm is terminated when the regression equations reach satisfactory levels. Otherwise, the residuals of X and Y after regression on *t*_1_ are used to extract the next principal component. The algorithm iterates until the results reach satisfactory levels.

Cross-validation (Q_h_^2^) is used as the measurement criterion to determine whether the regression results reach the satisfactory level. For the number of extracted principal components h, rounding observation i (i = 1,2, ⋯, n) for each time, (i = 1,2, ⋯, n), the PLS model is built with the remaining (n−1) observations. Then, observation i is substituted in the fitted PLS regression equation to obtain the predicted value of y_j_ (j = 1,2, ⋯, q) at observation i, and the predicted value is recorded as $\hat{y_{(i)j}}(h)$. The above calculation is repeated for each i (i = 1,2, ⋯, n). The sum of the squared errors (SSE) for dependent y_j_ is obtained when h principal components are extracted and ${PRESS}_{j}(h)=\sum_{i=1}^{n}{\left(y_{ij}-\hat{y_{(i)j}}(h)\right)}^{2}$ is recorded. Then, the sum of SSE for Y = [y_1_, y_2_, ⋯, y_q_] is obtained and $PRESS(h)=\sum_{i=1}^{q}{PRESS}_{j}(h)$ is recorded. All observations are likewise used to fit the regression equation with h principal components. At this time, the prediction value for observation i is noted as $\hat{y_{(i)j}}(h)$. The sum of SSE for y_j_ is defined as ${SS}_{j}(h)=\sum_{i=1}^{n}{\left(y_{ij}-\hat{y_{(i)j}}(h)\right)}^{2}$, and the sum of SSE for Y is defined as $SS(h)=\sum_{j=1}^{q}{SS}_{j}(h)$. Cross-validation is defined as *Q*_*h*_^2^ = 1 − *PRESS*(*h*)/*SS*(*h* − 1) Thus, a cross-validation test is performed before the end of each modelling step. The model estimation reaches a satisfactory level of precision and the extraction of components is stopped if *Q*_*h*_^2^ < 1 − 0.95^2^ = 0.0975 is satisfied at step h. If *Q*_*h*_^2^ ≥ 0.0975 is satisfied at step h, then the marginal contribution of the extracted principal component t_h_ is significant, and step (h+1) should be calculated.

After m principal components t_1_, t_2_, ⋯, t_m_ are finally extracted from X, PLS first performs a regression of y_k_ on t_1_, t_2_, ⋯, t_m_ and converts it in the regression equation of y_k_ on *x*_1_, *x*_2_, ⋯, *x*_p._

The specific procedures of the PLS algorithm are summarised as follows:

**Step 1.** To simplify the calculation and eliminate the effects of different units of variables, this study first standardises the original data matrices (X and Y), which are denoted by E_0_ and F_0_.
**Step 2.** Let t_1_ be the first principal component extracted from E_0_. The regression of E_0_ and F_0_ on t_1_ is performed as follows:

$$E_{0}=t_{1}p_{1}^{\prime }+E_{1},F_{0}=t_{1}r_{1}^{\prime }+F_{1}.$$

Where p_1_ and r_1_ refer to regression coefficient vectors, and E_1_ and F_1_ represent the corresponding residual matrices. The accuracy of the regression equation is calculated. The algorithm is terminated when the regression equations reach satisfactory levels. Otherwise, let E_0_ = E_1_ and F_0_ = F_1_, and iterate the component extraction and regression analysis. Cross validation (Q_h_^2^) is used to evaluate the model until the expected accuracy is obtained.

**Step3.** The number of regression components should be selected. The number of regression components included in the PLS model is important because it directly affects the fitting accuracy of the model. It should be carefully selected based on cross validation (Q_h_^2^). If Q_h_^2^ is higher or equal to 0.0975, then the marginal contribution of component t_h_ is significant and contributes to the precision of estimation results.
**Step4.** The regression equation of E_0_ and F_0_ on t_1_, t_2_, ⋯, t_m_ is derived if the model extracts m principal components. The following regression equation is developed through inverse transformation.

In the calculation of PLS, the principle component t_h_ should both represent the variation information of X (x_j_ (j = 1, 2, …, p)) and explain the information of Y (y_k_ (k = 1, 2, ⋯, q)) as much as possible. To measure the explanatory power of t_h_ for interpreting X and Y, we define various explanatory powers of t_h_ as follows:

1. The explanatory power of t_h_ to interpret X: $Rd(X;t_{h})=\frac{1}{p}\sum_{j=1}^{p}Rd(x_{j};t_{h})$;
2. The cumulative explanatory power of t_1_, t_2_, ⋯, t_m_ to interpret X: $Rd(X;t_{1},t_{m})=\sum_{h=1}^{m}Rd(X;t_{h})$;
3. The explanatory power of t_h_ to interpret Y: $Rd(Y;t_{h})=\frac{1}{q}\sum_{k=1}^{q}Rd(y_{k};t_{h})$;
4. The cumulative explanatory power of t_1_, t_2_, ⋯, t_m_ to interpret Y: $Rd(Y;t_{1},t_{m})=\sum_{h=1}^{m}Rd(Y;t_{h})$.

A significant advantage of PLS regression is the reliable choice of variables. When independent variable x_j_ is used to explain the set of dependent variables Y, the variable importance in projection VIP_j_ can be used to measure the importance of x_j_ in interpreting Y [69].

The expression of VIP_j_ is ${VIP}_{j}=\sqrt{\frac{p}{Rd(Y;t_{1},t_{m})}\sum_{h=1}^{m}Rd(Y;t_{h}){w^{2}}_{hj}}$, where p represents the number of independent variables, and w_hj_ is the linear combination coefficient of the principal component t_h_. For principle component t_h_, t_h_ = w_h1_x_1_+w_h2_x_2_+⋯+w_hp_x_p_. For h = 1,2, ⋯,m, $\sum_{j=1}^{p}{w^{2}}_{hj}=1$. The explanatory power of x_j_ to Y is transferred by t_h_. Formula ${VIP}_{j}^{2}=\frac{p\sum_{h=1}^{m}Rd(Y;t_{h}){w^{2}}_{hj}}{\sum_{h=1}^{m}Rd(Y;t_{h})}$ indicates that when the values of Rd(Y;t_h_) and w^2^_hj_ are large, VIP^2^_j_ will also gain a large value.

Formula $\sum_{j=1}^{p}{VIP}_{j}^{2}=\sum_{j=1}^{p}\frac{p\sum_{h=1}^{m}Rd(Y;t_{h}){w^{2}}_{hj}}{\sum_{h=1}^{m}Rd(Y;t_{h})}=\frac{p\sum_{h=1}^{m}Rd(Y;t_{h})}{\sum_{h=1}^{m}Rd(Y;t_{h})}\sum_{j=1}^{p}{w^{2}}_{hj}=p$ indicates that if the VIP_j_ of all independent variables x_j_(j = 1,2, …,p) equals 1, then they all play the same role in interpreting Y. Otherwise, x_j_ exerts a significant effect on interpreting Y when VIPj> 1.

## Results and discussion

### Seasonal variation characteristics of PM_2.5_ concentration

**Fig 7:**
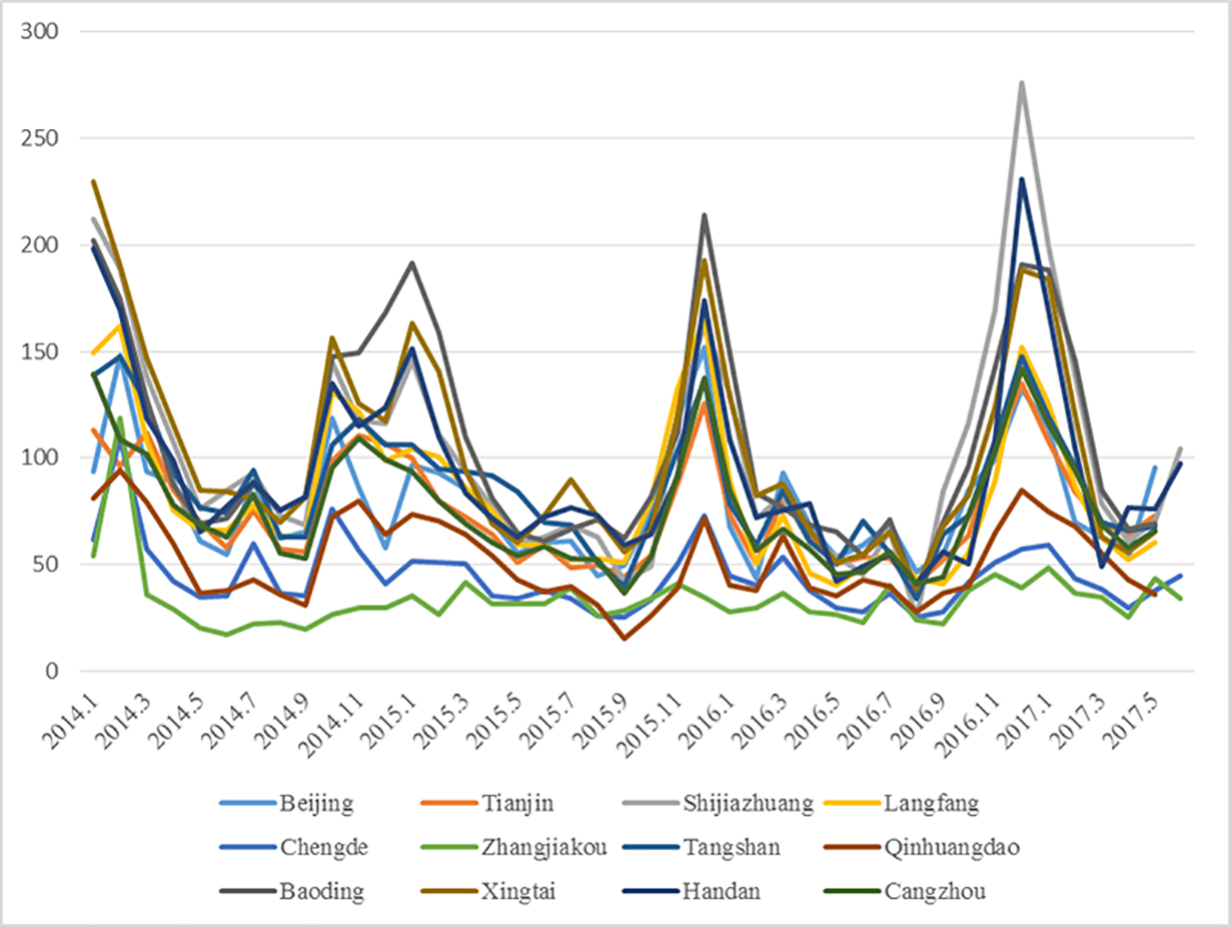
Seasonal variations of PM_2.5_ concentration of 12 cities in the BTH urban agglomeration (Unit: μg/m^3^).

The average PM_2.5_ concentration in the BTH urban agglomeration shows a notable periodical U-shaped variation from December 2013 to May 2017. The annual average PM_2.5_ concentration in all cities is 77.79 mg/m^3^. The monthly variation of PM_2.5_ concentrations in the BTH region (Fig 7) shows that the PM_2.5_ concentration is below 35 mg/m^3^ for only 8.3% of the time (Interim target-1 of WHO, 2005). Specifically, PM_2.5_ concentration is high in autumn and winter and low in spring and summer. This finding is consistent with other findings on China’s PM_2.5_ [51, 66, 70]. PM_2.5_ concentration from January to May shows a downward trend and from June to September maintains a stable level at 35–65 μg/m^3^, which is slightly lower than those from January to May. PM_2.5_ concentration from October to December exhibits an upward trend, with an increase from 58.95 μg/m^3^ to 144.8 μg/m^3^. The highest average PM_2.5_ concentration occurs in February 2014 at 142.43 μg/m^3^, whereas the lowest average PM_2.5_ concentration occurs in August 2016 at 35.46 μg/m^3^. The highest PM_2.5_ concentrations are recorded in December in Shijiazhuang (276.30 mg/m^3^), whereas the lowest PM_2.5_ concentrations are recorded in September in Qinhuangdao (14.90 mg/m^3^). Therefore, a high PM_2.5_ concentration condition generally occurs from October to January, especially in December. The change in PM_2.5_ concentration every year is regular from January to December. Estimation results in the BTH urban agglomeration are consistent with observations.

Interpolation results add evidence that PM_2.5_ concentration in the BTH urban agglomeration exhibits a pronounced characteristic of seasonal variation. From December 2013 to May 2017, PM_2.5_ concentration is significantly high in autumn and winter and low in spring and summer. Coal burning in Northern China for winter warming can be expected to be the main contributor to the highest concentration of PM_2.5_ in the country [20, 22, 27, 28]. Average PM_2.5_ concentration measures 135.6 μg/m^3^ in winter and 64.1 μg/m^3^ in summer, and the total mean values of PM_2.5_ in spring and autumn are 81.5 and 89.6 μg/m^3^, respectively.

Related studies have recorded that coal consumption for heating in autumn and winter is the main reason for the difference in values [66]. Coal combustion remains the main way of heating for residents in winter. Thus, government in the BTH regions are proactively promoting coal substation schemes, such as urban gasification projects. Coal is ‘dirty’ energy, and emissions of particle pollutants are enormous in winter. Meteorological factors also contribute to high PM_2.5_ in winter [39]. Specifically, rainfall in winter is scarce relative to the other seasons, the flushing effect of rainfall to air is little, inhalable particles are easily suspended in air and low temperature in winter is not conducive to the diffusion of PM_2.5_ particles.

**Fig 8:**
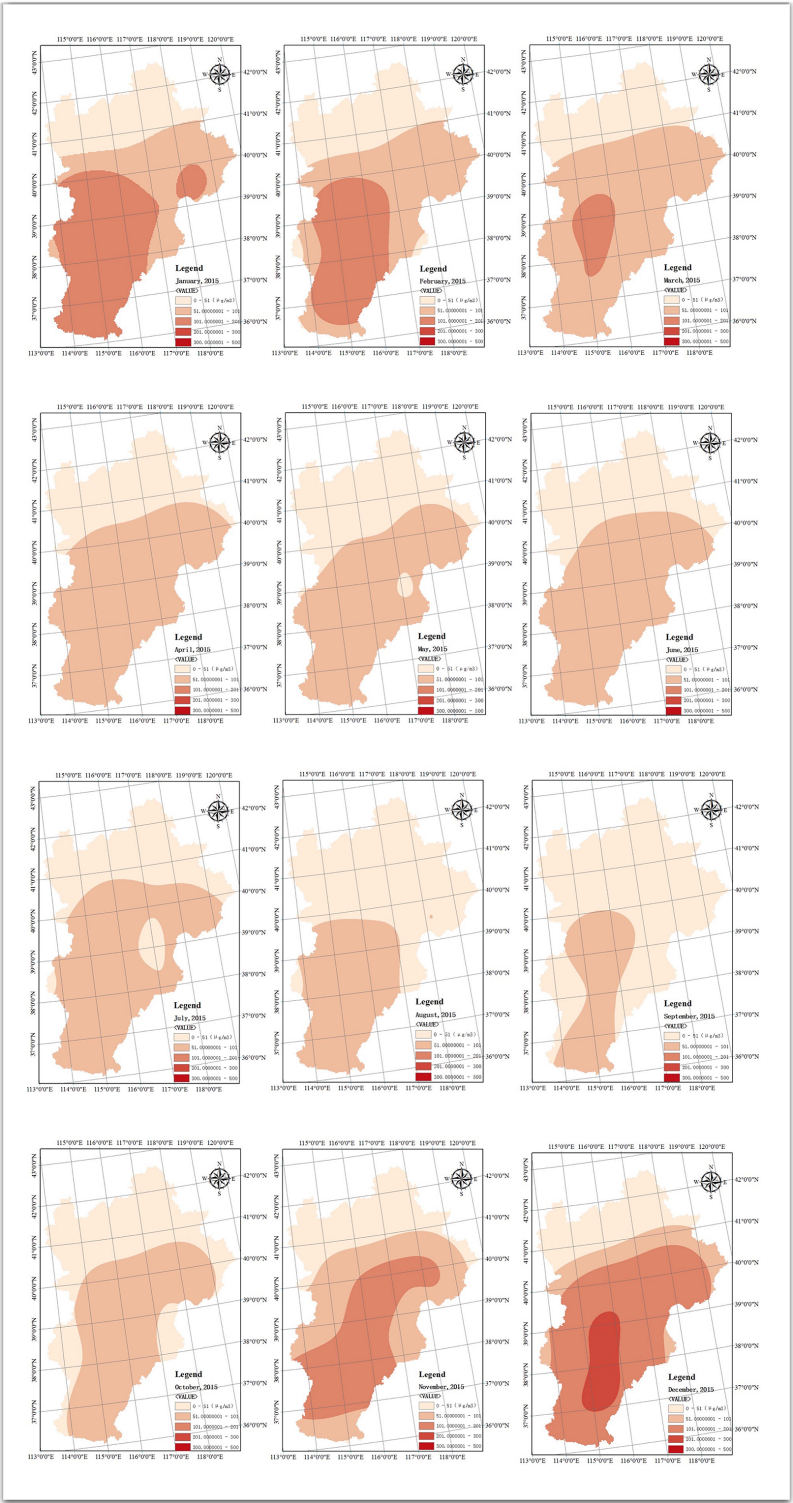
Spatial–temporal variation of PM_2.5_ concentration from January 2015 to December 2015 (Unit: μg/m^3^).

This study employed OKM to analyse the seasonal variation of PM_2.5_ concentration of 12 cities in the BTH urban agglomeration from December 2013 to December 2014. The Ministry of Environmental Protection of the People’s Republic of China (2018) [38] categorises PM_2.5_ concentration into five classes: (1) 0–50 ug/m^3^, (2) 50–100 ug/m^3^, (3) 101–200 ug/m^3^, (4) 200–300 ug/m^3^ and (5) 300–500 ug/m^3^. Fig 8 shows that PM_2.5_ concentration for every month is below the fourth classification of 200–300 ug/m^3^. The most heavy air pollution areas are recorded in the southern and eastern parts of the BTH region, especially Shijiazhuang City.

### Spatial variation characteristics of pm_2.5_ concentration

**Fig 9:**
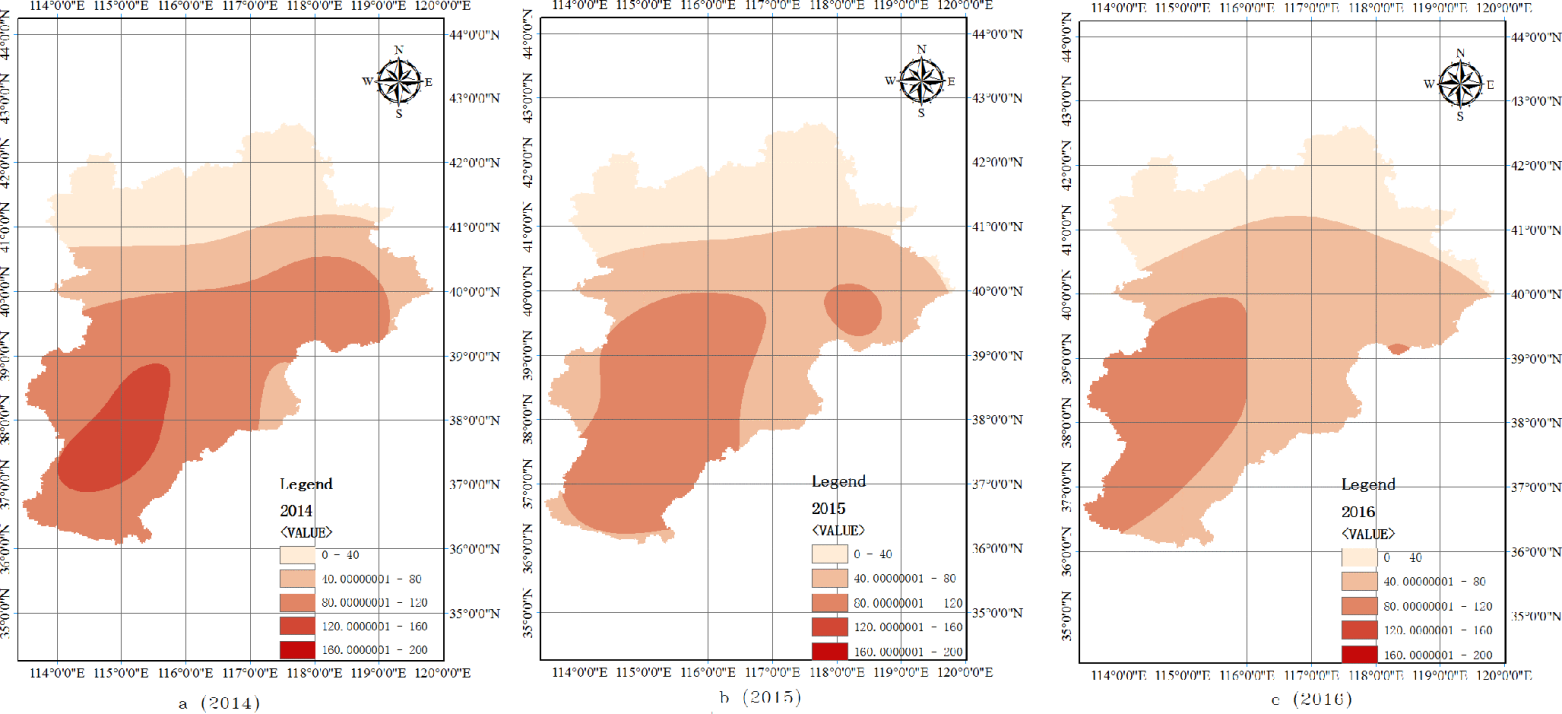
**Spatial variation of PM_2.5_ concentration in 2014, 2015 and 2016 (a, b and c) (Unit: μg/m^3^)**.

PM_2.5_ concentration in the BTH urban agglomeration shows a significant spatial variation (Fig 9), which is also recorded in some studies [51, 52, 70]. From 2014 to 2016, PM_2.5_ concentration is significantly high in the south and east of BTH urban agglomeration, particularly in Shijiazhuang. The mean value of PM_2.5_ concentration in Shijiazhuang amounts to 104 μg/m^3^. PM_2.5_ concentration is significantly low in the north, particularly in Zhangjiakou. The mean value of PM_2.5_ concentration in Zhangjiakou measures 34 μg/m^3^. From 2014 to 2016, the annual PM_2.5_ concentration in the BTH urban agglomeration slightly decreases. By means of 3km satellite aerosol optical depth (AOD) database and geographically and temporally weighted regression (GTWR) modelling, He et al., (2018) [66] also add evidence to the fact that air pollution in the BTH urban agglomeration generally showed a decreasing trend from January 2013 to December 2015.

**Fig 10:**
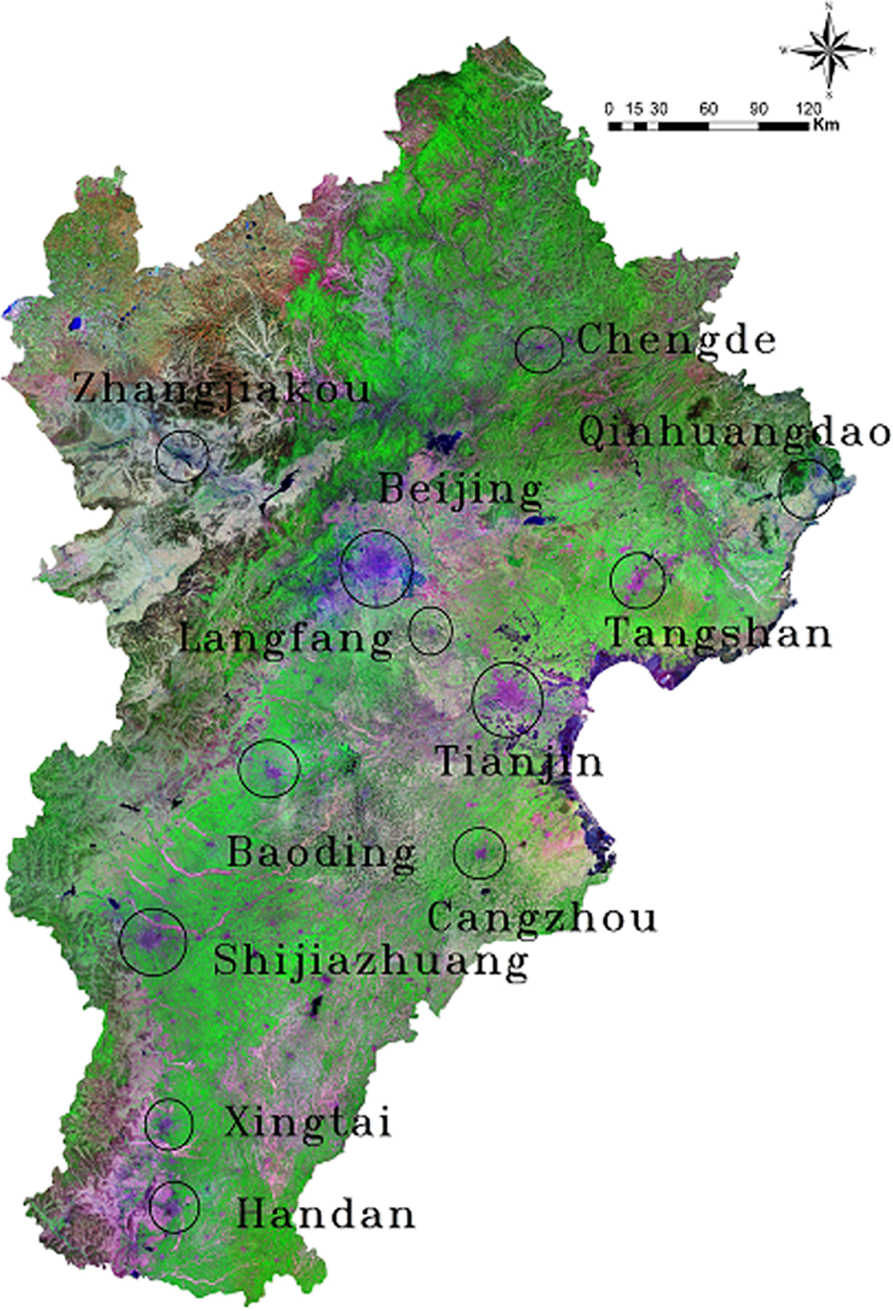
Remote sensing of BTH.

As shown in Fig 10, the remote sensing data is used to analyse the relationship between PM_2.5_ concentration and population density, landform, geography and elevation. Remote sensing data (Fig 10) shows that PM_2.5_ concentration is low in mountainous and basin regions, such as Zhangjiakou and Chengde, which are situated more than 1000 feet above sea level. High sea level is an advantageous landform for blocking the invasion of PM_2.5_ from peripheral regions [71]. Regions at high sea levels are also subject to northern and northwest winds, which benefit the dissemination of PM_2.5_ pollutants.

### Estimation results of PLS regression

Two key plots are considered when examining the performance of PLS regression. The first is t_1_/t_2_ oval plot.

**Fig 11:**
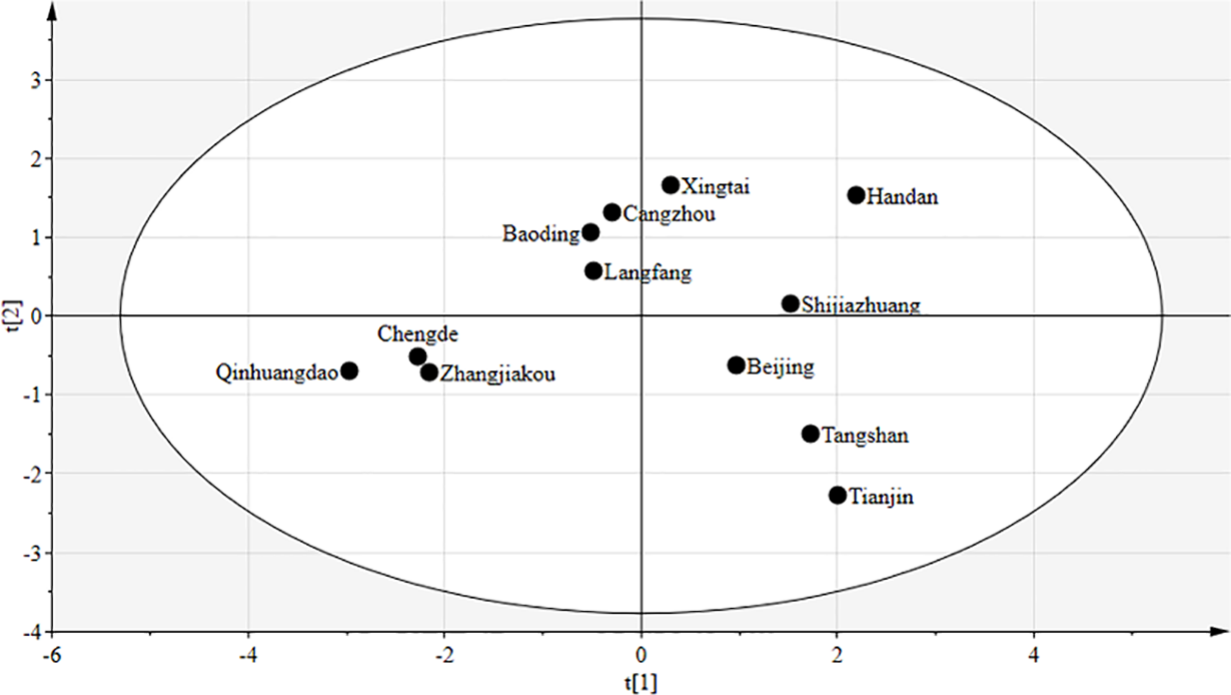
t_1_/t_2_ oval plot.

As the first two extracted linear combinations of x_1_, x_2_, …, x_p_, t_1_ and t_2_ represent the key information of X variables and exhibit remarkable explanatory power for Y variables [71,72]. The underpinning logic is straightforward: when all t_1_/t_2_ points are covered in the oval, raw data are homogenous and appropriate for model calculations [72]. Fig 11 shows that all the 12 observations of sample points are covered in the oval, indicating that the PLS model is suitable for use in this study.

**Fig 12:**
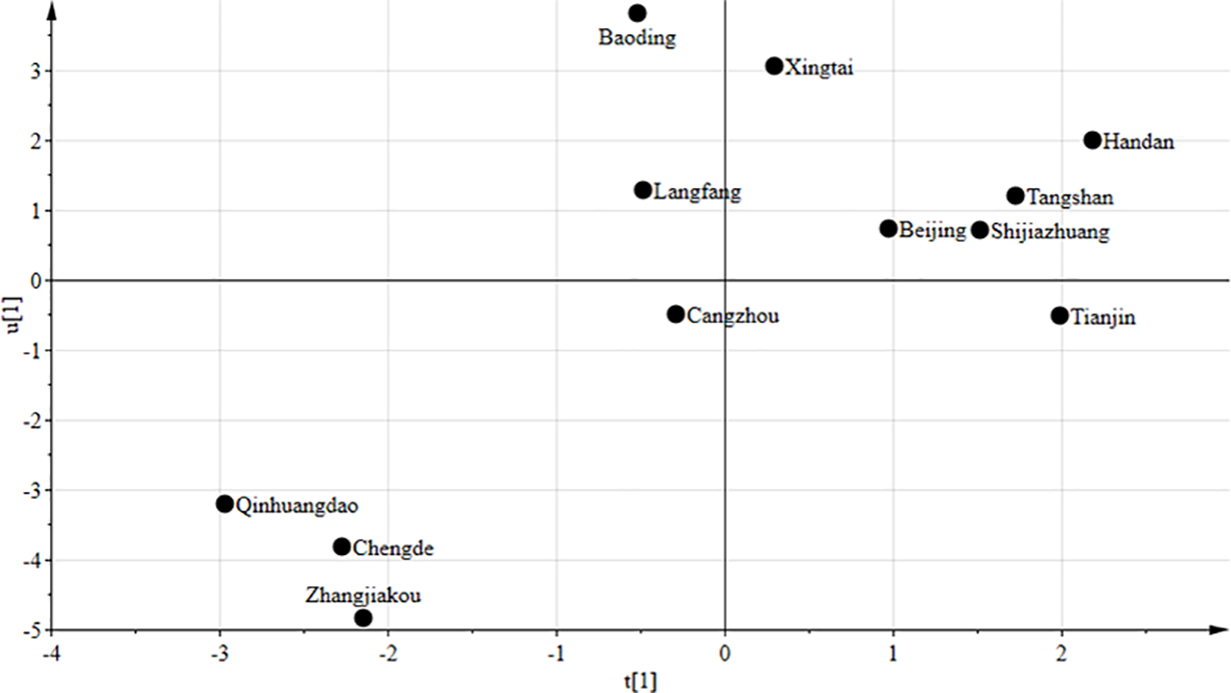
t_1_/u_1_ scatter plot.

Another plot that should be considered is the t_1_/u_1_ scatter plot. When t_1_/u_1_ of the sample data shows a nearly linear relationship, PLS is appropriate for studying the issue [73]. As shown in Fig 12, the scattering of sample data generally shows a linear relationship. Thus, the PLS model is suitable for use in this study.

Table 4 illustrates the overview results of PLS regression. The PLS regression is estimated by using SIMCA-P (a data analytic software). Two principle components are extracted according to the value of cross validation. When two components are extracted, ‘R^2^X(cum) = 0.510’ indicates that the two components exhibit explanatory powers for 51.0% of the variance of independent variables. ‘R^2^Y(cum) = 0.681’ indicates that the two extracted components explain 68.1% of the information on dependent variables, indicating the acceptable explanation power of the PLS method.

**Table 4:** Overview of PLS regression results.

Number of components	R^2^X(cum)	R^2^Y(cum)
1	0.339	0.424
2	0.510	0.681

VIP is a critical parameter for measuring the fitting performance of the PLS model [33, 69]. It quantifies the statistical significance of independent variables (X) in explaining dependent variables. When the VIP value of a variable is higher than 0.8, then the variable is ‘important’ and exhibits significant explanatory powers on the independent variables [69]. Table 5 presents the significance of 12 independent variables in evaluating the PM_2.5_ index amongst 12 cities of the BTH region.

**Table 5:** VIP values of factors.

Abbreviation	Corresponding variable	VIP value
**PD**	Population density	1.774
**UR**	Urbanisation rate	1.476
**RFV**	Road freight volume	1.157
**SIGDP**	Secondary industry GDP	0.953
**OEC**	Overall energy consumption	0.890
**SP**	Steel production	0.889
**VOSDE**	Volume of Sulphur Dioxide Emission	0.864
**VOISE**	Volume of Industrial Soot(dust) Emission	0.784
**CP**	Cement production	0.762
**MVO**	Motor vehicle ownership	0.749
**RPTV**	Road passenger traffic volume	0.504
**RNGC**	Residential natural gas consumption	0.295

Table 5 shows that the VIP values of most variables reach more than 0.8, that is, most variables are significant in explaining the PM_2.5_ concentration. The VIP values of population density, urbanisation rate and road freight volume are higher than 1.0. These results indicate that the variables are the three top contributors to PM_2.5_ concentration in the 12 sample cities of the BTH urban agglomeration. The VIP values of secondary industry GDP, overall energy consumption, steel production and volume of sulphur dioxide emission are higher than 0.8. Therefore, these factors are also major drivers of PM_2.5_ pollutant emission. The rest of the variables with VIP values in the spectrum of 0.5–0.8 cannot be assessed or are unimportant drivers of pollutant emission [72].

**Fig 13:**
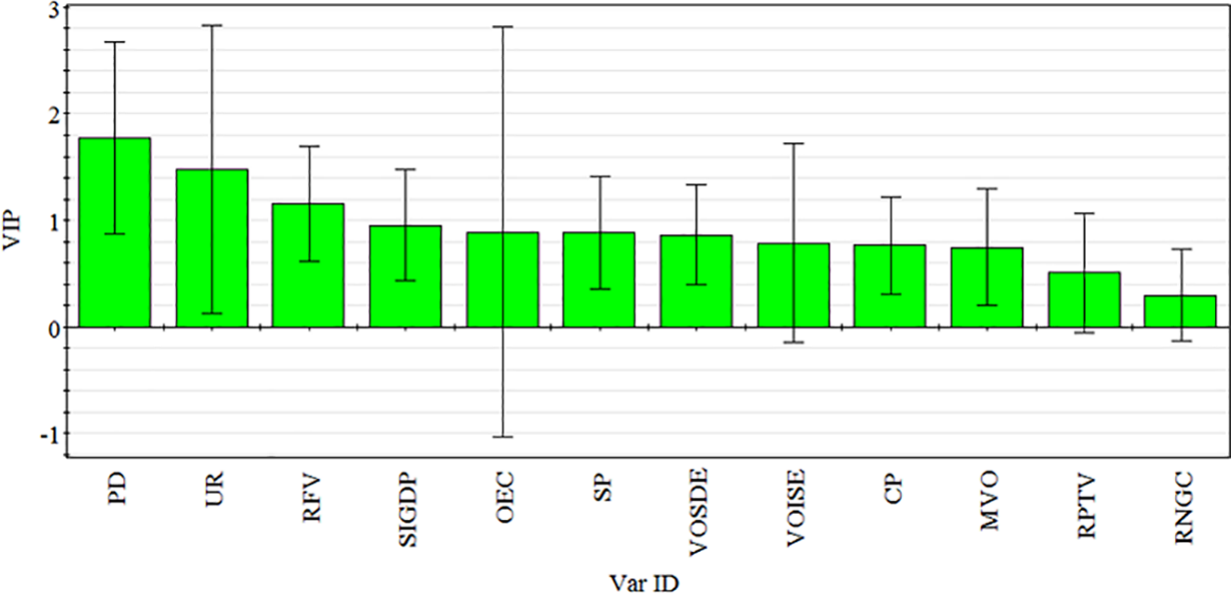
Variable importance plot.

The VIP value of population density is 1.774, which is the highest explanatory power for PM_2.5_ concentration. The VIP value of urbanisation rate in explaining PM_2.5_ concentration is 1.476, which ranks second, as shown in Fig 13. Previous studies have demonstrated that PM_2.5_ concentration is particularly high in large cities and urbanised regions [1, 2, 4, 5]. BTH is one of the most urbanised, populous and developed urban agglomerations in China, and human activities, such as transport, productions of secondary industry and energy consumption, are intensive in this region. Active human activities demand considerable resource and energy and create significant traffic daily. Numerous studies have reported that vehicular exhaust is the main source of PM_2.5_ [61]. Coal and oil, as cost-effective energy, have long been used as main fuels for the secondary industry in developing countries [30]. In particular, in industrial zones of BTH, coal consumption is the main fuel for energy- and emission-intensive sectors, such as those of steel, cement and glass, which are key materials for China’s remarkable infrastructure build-up in the past decades. Coal combustion is the main air pollution source in this region [59].

The VIP value of road freight volume totals 1.157 in explaining PM_2.5_ concentration. Previous studies have demonstrated that road vehicular exhaust is highly related to PM_2.5_ concentration [26, 29, 62, 63]. According to literature, road freight volume, particularly those of heavy-duty trucks, is the main air pollution source in the transportation sector in some cities [64]. However, in the current study, the VIP value of road passenger traffic volume reaches only 0.504, which is much less than that of road freight volume. Therefore, this factor is not a primary air pollutant source.

The VIP value of the secondary industry GDP totals 0.953 in explaining PM_2.5_ concentration. In the industrial zones of BTH, such as Tangshan, coal is the main energy in different sectors because of their copious coal deposits. However, carbonaceous aerosols are the main source of PM_2.5_. Coal combustion and sulphur dioxide are the dominant sources of carbonaceous aerosols [61]. Secondary industry, heating for residences in winter and electricity from coal-fired stations rely heavily on coal combustion [20, 22, 27, 28]. Thus, the VIP value of overall energy consumption in explaining PM_2.5_ concentration is high at 0.890. The VIP value of steel production in BTH in explaining PM_2.5_ concentration is 0.889, because metal elements correspond to other sources of PM_2.5_ in China. The process of producing steel requires significant coal combustion and releases abundant metal elements and sulphur dioxide into air [61]. Studies have recorded that PM_2.5_ concentrations exhibit a notable and positive relationship with associated air pollutants, such as SO_2_, NO_2_ and O_3_, and suggested that those atmospheric pollutants can evolve from primary pollution to secondary pollution and form a vicious cycle [39, 74].

As China’s political and economic centre, the particulate matter (PM) pollution in the Beijing-Tianjin-Hebei (BTH) urban agglomeration attracts extensive attention of the scholars. Many recent studies investigate PM_2.5_ concentration characteristics in the BTH region by using various methods and data sources. Similarities and new evidences of empirical findings of this study are compared with those of related studies.

First, some findings of this research are generally consistent with conclusions of existing studies. For example, this study reconfirmed that the annual PM_2.5_ concentration experienced a slight downturn in recent years, which is also recorded by He et al (2018) [67]. For temporal characteristics of PM_2.5_ concentration, the study captured a periodic U-shaped variation pattern in BTH urban agglomeration with high pollution levels in autumn and winter and low levels in spring and summer. Yan et al. (2018) [51] also recorded a pronounced characteristic of seasonal variation. They found that the concentrations increased from late autumn to early winter and that the PM_2.5_ concentration decreased rapidly from late winter to early spring. For spatial aspects, empirical results of this study find that the south and east of the BTH urban agglomeration where are densely populated are suffered with highest PM_2.5_ concentration and that PM_2.5_ concentration is significantly low in the north. Similar spatial characteristics are also recorded in existing studies [52, 67].

Second, this study employed data with a long period from December 2013 to May 2017, which ensures robust and complete understandings on PM_2.5_ concentration characteristics in the region. Most studies on the BTH urban agglomeration used either one year cross-sectional data or daily time series data [50, 51, 70] or outdated data before 2015 [67]. For example, based on daily monitoring data from 1 January 2014 to 31 December 2014, Liu et al. (2018) [50] investigated dynamic interactions and relationships between PM_2.5_ concentrations in different cities. Estimation results based on a short period of data undermined the understandings on the temporal characteristics of PM_2.5_. In addition, since 2015, Chinese government has committed great efforts and resources in PM_2.5_ treatment and special focuses are dedicated in the BTH region. Findings with data before 2015 cannot reflect the recent characteristic of PM_2.5_ concentrations and effectiveness of PM_2.5_ treatment.

Third, this research combines the satellite sensing data and monitoring sites data. Some academics carried out their studies based on satellite sensing data [52,67,75], meanwhile others used the data obtained from surface monitoring stations [51, 70]. In this study, the surface monitoring data of 12 cities in the regions are measured by 80 monitoring stations distributed throughout the BTH region, which is used for statistical modeling. In addition, remote sensing data is processed by OKM method to uncover the temporal and spatial characteristics. The remote sensing data are also used to analyze the relationship between PM_2.5_ concentration and landform, geography and elevation. Remote sensing data (Fig 10) shows that PM_2.5_ concentration is low in mountainous and basin regions, which are situated more than 1000 feet above sea level.

Fourth, this study focused on the driving factors of PM_2.5_ concentration, besides one goal of investigating the temporal and spatial characteristics. Related studies on the BTH regions are generally salient on diving facts of PM_2.5_ concentration. For example, Zheng et al. (2018) [70] focused on improvement of the real-time forecast. Liu et al.(2018) [50] aim to visualize the dynamic interactions and relationships between PM_2.5_ in different cities in the BTH regions. Yan et al. (2018) [51] investigated the spatiotemporal pattern of PM_2.5_ concentrations in China. In this study, a large number of socioeconomic factors are included in the investigation. Partial least squares (PLS) method to explore the critical driving factors of PM2.5, which is rarely used in PM_2.5_ research. Estimation bias caused by multicollinearity of raw data can be avoided and it can lead to a robust estimation results. Empirical results demonstrate that the deterioration of PM_2.5_ concentration in 2015 is closely related to a set of critical impact factors, including population, transport, industry production, and energy consumption aspects.

## Conclusion

PM_2.5_ is a challenging and urgent air pollutant that should be fully treated in China. The BTH region is heavily exposed to serious PM_2.5_ pollution. Previous studies are limited by the lack of real-time monitoring data and poor consideration of multicollinearity issues amongst dependent variables. This study aims to quantitatively measure the spatial–seasonal concentration characteristics of PM_2.5_ and identify critical impact factors. Empirical findings reveal the following. (1) Notable differences in PM_2.5_ concentrations exist amongst different seasons. Specifically, a periodical U-shaped variation trend with high pollution levels is found in autumn and winter and one with low levels is observed in spring and summer. (2) An apparent spatial distribution pattern exists in which PM_2.5_ concentration in the south is higher than in the northern regions in BTH. (3) The deterioration of air pollution is closely related to several critical impact factors, including population density, urbanisation rate, road freight volume, secondary industry GDP, overall energy consumption and industrial pollutants (e.g. steel production and volume of sulphur dioxide emission).

Numerous viable and concrete police recommendations are provided for effective PM_2.5_ treatment. 1) Decentralisation policy is a viable alternative policy for improvement of PM_2.5_ treatment. Decentralisation policy is an effective approach for downsizing urban population and relieving congestion [76, 77]. Empirical studies verify a tight relation between population density and PM_2.5_ concentration. For example, rapid population influx in Beijing leads to PM_2.5_ concentration deterioration due to associated energy consumption and production. Population and emission-intensive industries should be decentralised outward under the cooperated plan of BTH integration. 2) Expansions of high-energy consumption and emission industries should be constrained. Non-coal or renewable energies, such as natural gas, solar, biomass and wide and regular hydro power, should be encouraged for wide commercialisation and deployment. Statuary standards for energy use efficiency and pollutant emission should be reinforced to raise the consciousness and capability of enterprises on PM_2.5_ treatments. 3) Mass transportation should be encouraged by the government and the public. For example, a complete and convenient mass transportation facility should be developed by the government to improve public ridership rate; the number of fuel-based motor vehicle ownership should be restricted; non-fuel cars, such as pure electric and plug-in new energy, should be encouraged; and diverse low-emission transportation modes, such as walking, cycling, car sharing and subway, should be used by urban residents. 4) Winter is the most heavily polluted season in the BTH region. Heating and vast coal combustion are associated, to a large extent, to contributing to high PM_2.5_ emission. In addition, meteorological conditions in winter are not conducive to the purification and diffusion of PM_2.5_ particles, thus adding to PM_2.5_ pollution. Ma and Zhang (2014) [16] emphasised that the wide use of imported low-calorie coal and lignite in industrial and domestic sectors are particularly adverse for treatments of PM_2.5_ pollution. The Chinese government should reduce the use of low-calorie coal and promote energy substations for clean heating energy. 5) PM_2.5_ pollution is more serious in the south than in the north. The government should formulate an industrial restructuring scheme to avoid excessive concentration of polluting industries in certain regions, plan scientific reallocation for highly polluting industries and upgrade the energy use efficiency of industrial sectors.

## Supporting information

S1 Table Geographic information of atmospheric physics observation points. The longitude and latitude information of 80 atmospheric physics observation points are reported.
(DOC)

S2 Table PM2.5 concentration data of 12 cities in the beijing-tianjin-hebei urban agglomeration. PM_2.5_ concentration data covers the period from December 2013 to May 2017 cover 12 cities.
(DOC)

S3 Table The possible critical impact factors of PM2.5 concentration in 2015. Consensus data of 12 potential contributing factors for PM_2.5_ concentrations are reported.
(DOC)
