# Supplementary material for: Spatial–seasonal characteristics and critical impact factors of PM2.5 concentration in the Beijing–Tianjin–Hebei urban agglomeration
Source: PLoS One. 2018 Sep 20;13(9):e0201364. doi: 10.1371/journal.pone.0201364 (PMC6147404; doi:10.1371/journal.pone.0201364)
Supplement: S1 Table — The longitude and latitude information of 80 atmospheric physics observation points are reported. (DOC) [file pone.0201364.s001.doc]

**S1 Table. Geographic Information of Atmospheric Physics Observation Points**

| **City** | **Observation Point** | **Longitude** | **Latitude** |
| --- | --- | --- | --- |
| Beijing | Wanshougong West | 116.3747278 | 39.88565298 |
| Beijing | Guan Park | 116.3693659 | 39.93736379 |
| Beijing | Ding Tomb | 116.2324668 | 40.30023118 |
| Beijing | Dong Si | 116.4350122 | 39.93865024 |
| Beijing | Temple of Heaven | 116.417313 | 39.88797708 |
| Beijing | Shunyi New City | 116.664263 | 40.17700804 |
| Beijing | Changping Town | 116.2330318 | 40.22994018 |
| Beijing | Nongzhanguan | 116.470307 | 39.94700908 |
| Beijing | Huairou Town | 116.6469836 | 40.30842471 |
| Beijing | Wanliu Haidian | 116.3056615 | 39.9727465 |
| Beijing | Gucheng | 116.1956398 | 39.91345006 |
| Beijing | Olympic Green | 116.403458 | 39.98966406 |
| Tianjin | Municipal inspection center | 117.1655924 | 39.10485468 |
| Tianjin | Train Factory | 117.360144 | 39.26002762 |
| Tianjin | Nanjing Road | 117.1944783 | 39.12627794 |
| Tianjin | Beichen Science Park | 117.2341735 | 39.23264805 |
| Tianjin | Ecology New City | 117.743989 | 39.12297614 |
| Tianjin | East River Station | 117.2586748 | 39.13502465 |
| Tianjin | Dongli School | 117.3159157 | 39.08910357 |
| Tianjin | West River Station | 117.2297745 | 39.11576308 |
| Tianjin | Tianshan Road | 117.2845218 | 39.14145262 |
| Tianjin | Taifeng Industrial park | 117.2161939 | 39.08871922 |
| Tianjin | Airport | 117.4346223 | 39.13485324 |
| **City** | **Observation Point** | **Longitude** | **Latitude** |
| Tianjin | Tuanpowa | 117.2241556 | 38.85470219 |
| Tianjin | Meijiang | 116.9778986 | 38.938618 |
| Tianjin | Yongming Road | 117.4707252 | 38.84471493 |
| Tianjin | Relay Factory | 117.193015 | 39.11961624 |
| Baoding | Huadian | 115.520781 | 38.8918471 |
| Baoding | Welcome Center | 115.4742448 | 38.91454898 |
| Baoding | Monitoring Station | 115.520822 | 38.87067098 |
| Baoding | Natatorium | 115.49179 | 38.86187012 |
| Baoding | Film Factory | 115.4589044 | 38.8789026 |
| Baoding | Water Works | 115.4491 | 38.95595 |
| Cangzhou | Environmental Protection Bureau | 116.8705601 | 38.32300189 |
| Cangzhou | TV transposer station | 116.8587151 | 38.3112269 |
| Cangzhou | City Building Department | 116.8853965 | 38.29896434 |
| Chengde | Detached Palace | 117.940068 | 41.00278794 |
| Chengde | China Bank | 117.9462307 | 40.9855138 |
| Chengde | Open Economic Zone | 117.9617875 | 40.92838757 |
| Chengde | Cultural Center | 118.176283 | 40.77135901 |
| Chengde | Railway Station | 117.9624 | 40.94407 |
| Handan | Sewage Disposal Work | 114.544662 | 36.61951091 |
| Handan | Environmental Protection Bureau | 114.5127094 | 36.6183012 |
| Handan | Congtai Park | 114.497151 | 36.62261394 |
| Handan | Kuangyuan | 114.4984 | 36.57512 |
| Hengshui | Environmental Protection Bureau | 115.6900555 | 37.7390633 |
| Hengshui | electric motor factory | 115.6933505 | 37.74699959 |
| Hengshui | Monitoring Station | 115.6417366 | 37.73790899 |
| **City** | **Observation Point** | **Longitude** | **Latitude** |
| Langfang | Environmental Protection Center | 116.7151272 | 39.55640104 |
| Langfang | Pharmaceutical Factory | 116.7140681 | 39.53533353 |
| Langfang | Open Economic Zone | 116.7641936 | 39.57409176 |
| Langfang | School of Aerospace Engineering | 116.74 | 39.52939 |
| Qinhuangdao | Monitoring Station | 119.6022102 | 39.95669165 |
| Qinhuangdao | Shanhaiguan | 119.7664087 | 40.01734233 |
| Qinhuangdao | Municipal Government | 119.6073071 | 39.94126759 |
| Qinhuangdao | Environmental Protection Bureau | 119.5257949 | 39.82796343 |
| Qinhuangdao | Jianshe Hotel | 119.1886195 | 39.72314183 |
| Shijiazhuang | Chemical Engineering School | 114.611311 | 37.99578903 |
| Shijiazhuang | Worker's Hospital | 114.5302533 | 38.0544777 |
| Shijiazhuang | Open Economic Zone | 114.6089161 | 38.04522071 |
| Shijiazhuang | The Northwest | 114.4716083 | 38.05681822 |
| Shijiazhuang | Fenglong Mountain | 114.359628 | 37.91493902 |
| Shijiazhuang | Hall of the People | 114.522283 | 38.05253405 |
| Shijiazhuang | Century Park | 114.537 | 38.02016 |
| Shijiazhuang | The Southwest | 114.4506 | 37.987 |
| Tangshan | Market | 118.204948 | 39.62617935 |
| Tangshan | Ceramics Factory | 118.17698 | 39.64441 |
| Tangshan | No.12 School | 118.179361 | 39.65621611 |
| Tangshan | Little Mountain | 118.310339 | 39.73518912 |
| Tangshan | Supply Bureau | 117.9701687 | 40.17602836 |
| Tangshan | Radar Station | 118.0224 | 39.72739 |
| Xingtai | Dahuoquan | 114.485547 | 37.09486498 |
| Xingtai | Normal School | 114.4551531 | 37.10855108 |
| **City** | **Observation Point** | **Longitude** | **Latitude** |
| Xingtai | Road&Bridge Corporation | 114.528402 | 37.09356294 |
| Xingtai | Environmental Protection Bureau | 114.489932 | 37.10734598 |
| Zhangjiakou | People's Park | 114.8958519 | 40.83098513 |
| Zhangjiakou | Probe factory | 114.8894659 | 40.7913336 |
| Zhangjiakou | Shijihaoyuan | 114.9033043 | 40.76626478 |
| Zhangjiakou | Beibengfang | 114.8872 | 40.80544 |
| Zhangjiakou | Wujinku | 114.8825 | 40.82162 |

Source: China Meteorological Administration
