# Supplementary material for: Spatial–seasonal characteristics and critical impact factors of PM2.5 concentration in the Beijing–Tianjin–Hebei urban agglomeration
Source: PLoS One. 2018 Sep 20;13(9):e0201364. doi: 10.1371/journal.pone.0201364 (PMC6147404; doi:10.1371/journal.pone.0201364)
Supplement: S2 Table — PM2.5 concentration data covers the period from December 2013 to May 2017 cover 12 cities. (DOC) [file pone.0201364.s002.doc]

**S1 Table. PM2.5 concentration data of 12 cities in the Beijing-Tianjin-Hebei Urban Agglomeration**

|  | Beijing’s | Tianjin’s | Shijiazhuang’s | Chengde’s | Zhangjiakou’s | Tangshan’s | Langfang ’s | Qinhuangdao ’s | Baoding ’s | Xingtai ’s | Handan ’s | Cangzhou ’s |
| --- | --- | --- | --- | --- | --- | --- | --- | --- | --- | --- | --- | --- |
| Month | PM_2.5_  (µg/m^3^) | PM_2.5_  (µg/m^3^) | PM_2.5_  (µg/m^3^) | PM_2.5_  (µg/m^3^) | PM_2.5_  (µg/m^3^) | PM_2.5_  (µg/m^3^) | PM_2.5_  (µg/m^3^) | PM_2.5_  (µg/m^3^) | PM_2.5_  (µg/m^3^) | PM_2.5_  (µg/m^3^) | PM_2.5_  (µg/m^3^) | PM_2.5_  (µg/m^3^) |
| 2013.12 | 73.3 | 120.3 | 164.8 | 47.3 | 40.2 | 135.4 | 143.8 | 67.4 | 189.2 | 181.6 | 199 | 133.5 |
| 2014.01 | 93.9 | 113 | 212.3 | 61.8 | 54.3 | 138.9 | 149.4 | 81.3 | 202.1 | 229.9 | 198.5 | 139.2 |
| 2014.02 | 148.4 | 96.3 | 189.1 | 109.4 | 118.5 | 147.5 | 162.1 | 94.4 | 174.8 | 190.2 | 169.6 | 108.9 |
| 2014.03 | 93.6 | 111.9 | 138 | 57.5 | 35.7 | 125.6 | 107.8 | 79.4 | 127.5 | 147 | 118.6 | 101.5 |
| 2014.04 | 88.6 | 84.6 | 107.3 | 42.3 | 29.1 | 92.9 | 75.1 | 59.9 | 87.2 | 115.6 | 98.8 | 78.1 |
| 2014.05 | 61.1 | 69.8 | 76.3 | 34.8 | 20.1 | 76.5 | 66.3 | 36.3 | 69.3 | 84.7 | 65.6 | 68.7 |
| 2014.06 | 54.4 | 58 | 84.7 | 35.4 | 17.2 | 74.3 | 66.3 | 37.7 | 71.9 | 84 | 77.4 | 62.9 |
| 2014.07 | 89.2 | 75.5 | 93.2 | 59.9 | 21.9 | 94.1 | 78 | 42.9 | 88 | 80.9 | 88.4 | 82.8 |
| 2014.08 | 62.3 | 56.9 | 72.8 | 36.2 | 22.6 | 62.8 | 63.7 | 35.6 | 75.5 | 69.7 | 75.6 | 55.2 |
| 2014.09 | 65.4 | 55.7 | 68.5 | 35.1 | 19.4 | 62.7 | 62.8 | 31 | 81.5 | 81.8 | 81.4 | 53.1 |
| 2014.10 | 118.5 | 98.9 | 145 | 76.2 | 26.2 | 106.2 | 131.2 | 72.3 | 147.8 | 156.2 | 134.9 | 95.7 |
| 2014.11 | 86.3 | 110.8 | 117.5 | 56.7 | 29.7 | 118 | 121.5 | 79.6 | 149.3 | 125.4 | 115.2 | 109.5 |
| 2014.12 | 57.8 | 106.7 | 116.4 | 41.1 | 29.6 | 106.3 | 98.9 | 64 | 168.1 | 117.3 | 123.8 | 99.2 |
| 2015.01 | 96.6 | 100 | 145.5 | 51.8 | 35 | 106.3 | 104.5 | 73.6 | 191.4 | 162.9 | 151 | 93.3 |
| 2015.02 | 92.9 | 79.5 | 111.4 | 51 | 26.2 | 95.1 | 100.3 | 70.6 | 159.1 | 140.9 | 110.2 | 79.6 |
| 2015.03 | 85.7 | 72.4 | 93.4 | 50 | 41.7 | 93.8 | 86 | 64.1 | 110.2 | 94.5 | 83.7 | 69.3 |
| 2015.04 | 70.8 | 64.4 | 77 | 35.4 | 31.8 | 91.7 | 75.2 | 53.8 | 80.8 | 69 | 71.5 | 60.6 |
| 2015.05 | 55.5 | 51.2 | 59.8 | 33.7 | 31.3 | 84 | 59.1 | 42.8 | 64.5 | 60.2 | 62.7 | 53.8 |
| 2015.06 | 60.3 | 59.3 | 63.1 | 37.7 | 31.5 | 69.9 | 58.8 | 37.3 | 60.9 | 73.1 | 72.2 | 58.4 |
| 2015.07 | 61.1 | 48.6 | 68.7 | 34.1 | 38.9 | 68.7 | 51.9 | 39.9 | 66.6 | 89.7 | 76.7 | 52.6 |
| 2015.08 | 44.9 | 49.9 | 62.8 | 25.7 | 25.7 | 51 | 52.8 | 30.9 | 71 | 72.1 | 73 | 51.9 |
| 2015.09 | 49.9 | 44.2 | 42.3 | 25.5 | 28.3 | 39.6 | 50.7 | 14.9 | 62.1 | 56.2 | 59 | 36.2 |
| 2015.10 | 74.1 | 53.9 | 49.3 | 33.5 | 33.7 | 70.1 | 79.9 | 25.8 | 81.5 | 65.9 | 64 | 53.2 |
| 2015.11 | 118.5 | 88.2 | 120.3 | 50.1 | 40.6 | 103.8 | 132.2 | 39.1 | 111.8 | 120.2 | 91 | 92.1 |
| 2015.12 | 151.7 | 125.3 | 162.8 | 72.9 | 34.8 | 136 | 165.6 | 71.7 | 214.1 | 192.8 | 174 | 137.6 |
| 2016.01 | 67.9 | 73.8 | 130.8 | 44.6 | 27.6 | 78.5 | 88.9 | 40.3 | 149.3 | 128.9 | 108.5 | 84.8 |
| 2016.02 | 43.5 | 50.2 | 71.4 | 40.4 | 29.5 | 59 | 50.6 | 38 | 83.4 | 82 | 72.1 | 56.4 |
| 2016.03 | 92.8 | 80.9 | 82.3 | 53.2 | 36.4 | 85 | 73.1 | 63.6 | 76.7 | 88.1 | 75.5 | 66.8 |
| 2016.04 | 68.8 | 63.5 | 60.3 | 37.7 | 27.5 | 61.2 | 46.2 | 39.3 | 68.5 | 66.2 | 78.6 | 57.3 |
| 2016.05 | 53.6 | 50.6 | 54.2 | 29.9 | 26.2 | 50.4 | 40 | 35.3 | 65.3 | 51.1 | 42.3 | 45.5 |
| 2016.06 | 59.4 | 53.5 | 45.6 | 27.6 | 22.8 | 70.1 | 49.1 | 42.9 | 54.3 | 54.4 | 49 | 46.6 |
| 2016.07 | 69.1 | 52.7 | 65.7 | 36.7 | 40 | 55.1 | 56.1 | 39.7 | 71 | 65.6 | 54.5 | 56.1 |
| 2016.08 | 46.6 | 40.8 | 25.2 | 25.5 | 24 | 34.3 | 43.2 | 27.8 | 37.7 | 38.8 | 40.9 | 40.8 |
| 2016.09 | 54.6 | 52.4 | 84.2 | 27.6 | 22.1 | 64.1 | 41 | 36.7 | 69.7 | 67.8 | 56 | 44 |
| 2016.10 | 84.6 | 63.7 | 116.1 | 41.6 | 37.6 | 73 | 55.1 | 39.6 | 96.1 | 82.3 | 50.6 | 72.6 |
| 2016.11 | 99.5 | 104.3 | 169.3 | 51.2 | 45.3 | 108.3 | 89.2 | 64.7 | 142.6 | 123.9 | 108.1 | 102.8 |
| 2016.12 | 132.7 | 135 | 276.3 | 57.2 | 39.2 | 147.5 | 151.7 | 84.7 | 190.6 | 188.3 | 230.7 | 142.1 |
| 2017.01 | 114.3 | 108.2 | 199.5 | 59 | 48.6 | 119.3 | 125.4 | 74.5 | 188.4 | 183.8 | 169.6 | 116.4 |
| 2017.02 | 69.9 | 84.1 | 138.8 | 43.3 | 36.3 | 95.9 | 86.8 | 67.8 | 145.4 | 120.6 | 104.6 | 94.1 |
|  | Beijing’s | Tianjin’s | Shijiazhuang’s | Chengde’s | Zhangjiakou’s | Tangshan’s | Langfang ’s | Qinhuangdao ’s | Baoding ’s | Xingtai ’s | Handan ’s | Cangzhou ’s |
| Month | PM_2.5_  (µg/m^3^) | PM_2.5_  (µg/m^3^) | PM_2.5_  (µg/m^3^) | PM_2.5_  (µg/m^3^) | PM_2.5_  (µg/m^3^) | PM_2.5_  (µg/m^3^) | PM_2.5_  (µg/m^3^) | PM_2.5_  (µg/m^3^) | PM_2.5_  (µg/m^3^) | PM_2.5_  (µg/m^3^) | PM_2.5_  (µg/m^3^) | PM_2.5_  (µg/m^3^) |
| 2017.03 | 62.7 | 69.8 | 78.6 | 38.4 | 34.9 | 69.7 | 62.6 | 55.3 | 84.9 | 63 | 49.3 | 68.5 |
| 2017.04 | 53.3 | 64.1 | 61 | 29.4 | 25.2 | 65.7 | 52.2 | 42.6 | 66.8 | 56 | 76.5 | 57.7 |
| 2017.05 | 95.7 | 72.7 | 70.1 | 37.7 | 43.3 | 67.6 | 60.5 | 36.1 | 68.9 | 65.6 | 76.2 | 65.9 |
