# Supplementary material for: Spatial–seasonal characteristics and critical impact factors of PM2.5 concentration in the Beijing–Tianjin–Hebei urban agglomeration
Source: PLoS One. 2018 Sep 20;13(9):e0201364. doi: 10.1371/journal.pone.0201364 (PMC6147404; doi:10.1371/journal.pone.0201364)
Supplement: S3 Table — Consensus data of 12 potential contributing factors for PM2.5 concentrations are reported. (DOC) [file pone.0201364.s003.doc]

| **S3 Table The possible critical impact factors of PM2.5 concentration in 2015** | | | | | | | | | | | | | |
| --- | --- | --- | --- | --- | --- | --- | --- | --- | --- | --- | --- | --- | --- |
| **Primary ID** | **PM2.5** | **RNGC** | **RFV** | **RPTV** | **PD** | **UR** | **SP** | **CP** | **SIGDP** | **OEC** | **MVO** | **VOSDE** | **VOISE** |
| **Unit** | (µg/m^3^) | 10000 cubic metres | 10000 tons | 10000 persons | Persons/KM^2^ | % | 10000 tons | 10000 tons | 100 millions | 10000 tons of standard coal | 10000 units | Ton | Ton |
| **Beijing** | 80.1667 | 189188 | 19044 | 49931 | 819.57 | 86.51 | 174.8 | 553.7 | 4526.4 | 6852.5 | 561.9 | 22070 | 12987 |
| **Tianjin** | 69.7417 | 28844 | 33724 | 14218 | 861.79 | 82.64 | 8186.16 | 777.59 | 7723.6 | 8078.04 | 509.29 | 154605 | 73795 |
| **Shijiazhuang** | 80.0333 | 8376 | 36954 | 5976 | 788.14 | 59.03 | 1060.34 | 2177.2 | 2452.9 | 2773.25 | 250.948 | 113652 | 87128 |
| **Langfang** | 84.75 | 2650 | 9918 | 3832 | 722.34 | 54 | 2315.62 | 918.16 | 1102.4 | 687.82 | 110 | 38390 | 48205 |
| **Chengde** | 41.7833 | 4579 | 4152 | 1163 | 96.73 | 46 | 1152.1 | 496.2 | 636.4 | 963.09 | 66.97 | 55393 | 50907 |
| **Zhangjiakou** | 33.2917 | 3134 | 11218 | 2081 | 127.46 | 52.2 | 597.47 | 458.12 | 545.53 | 1421.7 | 89.589 | 61858 | 35693 |
| **Tangshan** | 84.1667 | 7921 | 36358 | 2656 | 560.42 | 44.28 | 11179 | 2781 | 3365.4 | 7182.77 | 246.619 | 214723 | 46902 |
| **Qinhuangdao** | 47.0417 | 1482 | 5276 | 1924 | 379.39 | 47.53 | 574.18 | 259.31 | 445.09 | 430000 | 60.5621 | 46689 | 34702 |
| **Baoding** | 106.167 | 9820 | 10331 | 10792 | 541.81 | 23.48 | 31.85 | 727.04 | 1500.7 | 472.52 | 215 | 49850 | 31698 |
| **Xingtai** | 99.7917 | 5597 | 18711 | 3495 | 627.36 | 11.29 | 668 | 726.5 | 793.6 | 1168.84 | 141.89 | 76035 | 100738 |
| **Handan** | 90.75 | 666 | 38704 | 7129 | 870.29 | 16.74 | 5468.9 | 656.4 | 1500.7 | 3395.01 | 107.12 | 110193 | 191713 |
| **Cangzhou** | 69.8833 | 3236 | 19942 | 5473 | 551.48 | 7.1 | 2197.84 | 141.06 | 1602.5 | 5655.75 | 150 | 32712 | 50879 |
